# Generative whole-brain dynamics models from healthy subjects predict functional alterations in stroke at the level of individual patients

**DOI:** 10.1093/braincomms/fcae237

**Published:** 2024-07-13

**Authors:** Sebastian Idesis, Michele Allegra, Jakub Vohryzek, Yonatan Sanz Perl, Nicholas V Metcalf, Joseph C Griffis, Maurizio Corbetta, Gordon L Shulman, Gustavo Deco

**Affiliations:** Center for Brain and Cognition (CBC), Department of Information Technologies and Communications (DTIC), Pompeu Fabra University, Edifici Mercè Rodoreda, Barcelona, Catalonia 08005, Spain; Padova Neuroscience Center (PNC), University of Padova, Padova 35129, Italy; Department of Physics and Astronomy ‘G. Galilei’, University of Padova, 35131 Padova, Italy; Center for Brain and Cognition (CBC), Department of Information Technologies and Communications (DTIC), Pompeu Fabra University, Edifici Mercè Rodoreda, Barcelona, Catalonia 08005, Spain; Centre for Eudaimonia and Human Flourishing, Linacre College, University of Oxford, OX3 9BX, Oxford, UK; Center for Brain and Cognition (CBC), Department of Information Technologies and Communications (DTIC), Pompeu Fabra University, Edifici Mercè Rodoreda, Barcelona, Catalonia 08005, Spain; Universidad de San Andrés, Centro de Neurociencias Cognitivias, NC1006ACC, Buenos Aires, Argentina; National Scientific and Technical Research Council, C1425FQB, Buenos Aires, Argentina; Institut du Cerveau et de la Moelle épinière, ICM, Hôpital Pitié Salpêtrière, 75013 Paris, France; Department of Neurology, Washington University School of Medicine, St. Louis, MO 63110, USA; Department of Neurology, Washington University School of Medicine, St. Louis, MO 63110, USA; Padova Neuroscience Center (PNC), University of Padova, Padova 35129, Italy; Department of Neurology, Washington University School of Medicine, St. Louis, MO 63110, USA; Department of Neuroscience (DNS), University of Padova, Padova 35128, Italy; Department of Radiology, Washington University School of Medicine, St. Louis, MO 63110, USA; VIMM, Venetian Institute of Molecular Medicine (VIMM), Biomedical Foundation, Padova 35129, Italy; Department of Neurology, Washington University School of Medicine, St. Louis, MO 63110, USA; Department of Radiology, Washington University School of Medicine, St. Louis, MO 63110, USA; Center for Brain and Cognition (CBC), Department of Information Technologies and Communications (DTIC), Pompeu Fabra University, Edifici Mercè Rodoreda, Barcelona, Catalonia 08005, Spain; Institució Catalana de Recerca I Estudis Avançats (ICREA), Barcelona, Catalonia 08010, Spain

**Keywords:** whole-brain models, predictive, stroke, (f)MRI, dynamics

## Abstract

Computational whole-brain models describe the resting activity of each brain region based on a local model, inter-regional functional interactions, and a structural connectome that specifies the strength of inter-regional connections. Strokes damage the healthy structural connectome that forms the backbone of these models and produce large alterations in inter-regional functional interactions. These interactions are typically measured by correlating the time series of the activity between two brain regions in a process, called resting functional connectivity. We show that adding information about the structural disconnections produced by a patient’s lesion to a whole-brain model previously trained on structural and functional data from a large cohort of healthy subjects enables the prediction of the resting functional connectivity of the patient and fits the model directly to the patient’s data (Pearson correlation = 0.37; mean square error = 0.005). Furthermore, the model dynamics reproduce functional connectivity-based measures that are typically abnormal in stroke patients and measures that specifically isolate these abnormalities. Therefore, although whole-brain models typically involve a large number of free parameters, the results show that, even after fixing those parameters, the model reproduces results from a population very different than that on which the model was trained. In addition to validating the model, these results show that the model mechanistically captures the relationships between the anatomical structure and the functional activity of the human brain.

## Teaser

Idesis *et al*. showed that functional anomalies in stroke patients can be predicted mechanistically by adding only structural information concerning their lesion to a whole-brain computational model computed solely from healthy controls. Their results validated the integration of structural and functional information in whole-brain models of stroke.

## Graphical Abstract

**Graphical abstract:**
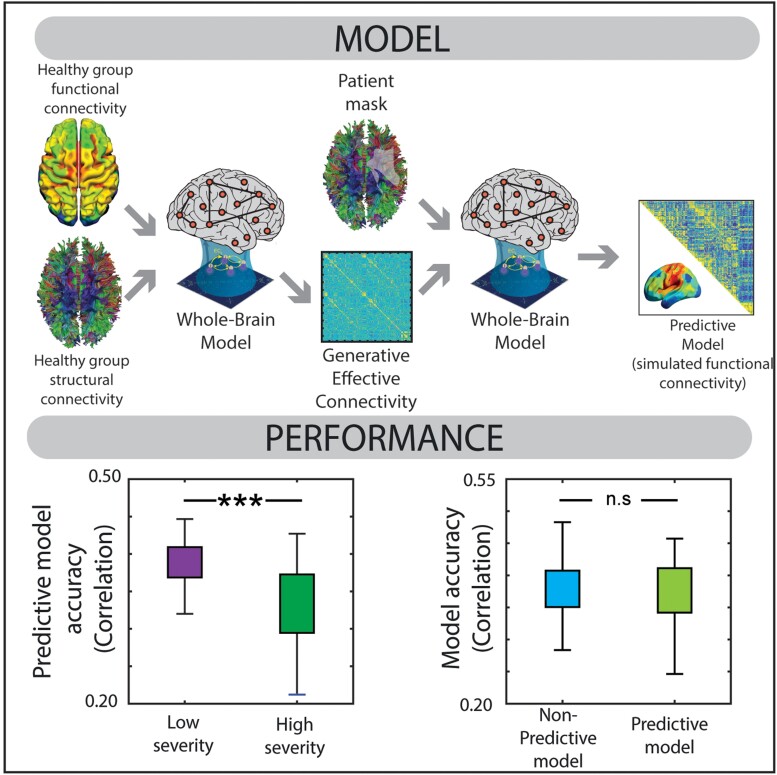

## Introduction

A significant development in neuroscience involves the conceptualization of the brain as a set of dynamic networks that interact and facilitate information processing through the integration and segregation of information. Correspondingly, the application of formal methods from the graph theory^1,2^ and the statistical mechanics for studying the structure and dynamics of those networks^3,4^ has been essential to this development. The spatiotemporal activity of the brain's resting-state physiology is identified by measuring the inter-regional correlation of the BOLD signal,^5^ so-called resting-state functional connectivity (FC). This spatiotemporal activity arises and is constrained by a structural connectome,^6^ a small-world organization,^7^ in which the ‘wiring efficiency’ is maximized by groups of densely interacting sets of brain regions (i.e. networks) that are linked by sparser connections. The structural organization of the brain generates spatiotemporal dynamics of activity or recurring waves across cortical, subcortical, and cerebellar circuits,^8^ which occur within a critical regime.^9,10^

The last decade has seen the development of computational models that simulate the spatiotemporal patterns of the brain activity by combining biologically plausible whole-brain descriptions of the macroscale structural connectivity with models of local regional activity.

These ‘whole-brain models’ have been able to replicate at least, at the group level, many of the spatial and temporal properties of the brain activity.^11^ Whole-brain models have also simulated activity changes produced by behavioural states, drugs or neurostimulation in the healthy or pathological brain.^12,13^

A key health-related application of whole-brain models is the simulation of the effects of brain pathologies, such as stroke, epilepsy or schizophrenia, on the brain activity and behaviour.^14,15^ Pathology-specific whole-brain models are very important because accurate simulations of the physiological effects of a pathology and their distributed impact on brain networks may not only provide insights into disease mechanisms, but may also additionally allow the effect of therapeutic interventions, such as drugs, rehabilitation or stimulation, to be modelled or predicted.^12^

Our group has pioneered the study of brain network alterations in stroke, and their simulation in whole-brain models. Focal lesions like stroke produce characteristic patterns of behavioural impairment and alterations in structural-functional connectivity measured with magnetic resonance imaging.^16–22^ In parallel, we and others have been able to show that whole-brain models can reproduce the abnormalities in network segregation, integration, variability and criticality of neural states that are observed following a stroke.^14,23–26^

However, whole-brain computational models often involve large numbers of free parameters, making validation a critical issue, which has been addressed using variants of out-of-sample prediction. In a leave-one-out procedure, the model is fit to all members of the sample, except for one, whose data are ‘predicted’. This procedure is then successively applied to each member of the sample. In a cross-validation procedure, the model is applied to a completely different sample from the same training population. However, a more demanding validation criterion would provide better support. Here, we fit the model to a sample from one population (i.e. healthy participants), and then apply it to a sample from a completely different population (i.e. stroke patients). ‘Out-of-population’ prediction can be implemented for a stroke population because the effects of a sub-acute stroke lesion on an important measure of the whole-brain function (i.e. resting-state FC) are primarily determined by the effect of the lesion on the brain’s structural connectome,^18^ and critically, the alterations in a patient’s structural connectome due to a lesion can be incorporated into the whole-brain model without introducing new parameters. In addition to validating the model, the application of the out-of-population procedure to stroke patients specifically tests how well the model integrates the functional brain activity with structural connectivity because the predicted physiology in a patient will only be accurate to the extent that the alterations in the structural connectivity produced by their lesion appropriately modify the model's outputs.

We apply this more demanding validation criterion to a whole-brain model that is generative (i.e. generates BOLD time series across a participant's brain). One advantage of a generative model is that it allows the prediction of any functional brain measure that can be computed from a BOLD time series (i.e. the model is not restricted to predicting FC).

Another noteworthy aspect of the generative, predictive whole-brain model of stroke that is evaluated in this paper is that it predicts measures of the brain function and behaviour for individual patients. Briefly, a ‘healthy’ model is first derived in a group of healthy control subjects by combining local, parcel-level measures of activity from a Hopf model^14,27^ with a population-level structural connectome^28^ that is modified during model fitting using directional connectivity parameters (GEC parameters) that instantiate generative effective connectivity. The resulting healthy model is then ‘damaged’ separately in each patient by using the structural disconnections produced by their lesion to proportionally modify the GEC parameters, a modification that introduces any new parameters. Finally, the model-derived activity time series for each patient is convolved with a hemodynamic response function to generate a blood oxygenation level-dependent (BOLD) time series across the brain. Previous stroke-related models optimized the patient fitting by including both the anatomical and functional information of each subject.^26^ In contrast, this generative, predictive whole-brain model satisfies an out-of-population validation criterion because it does not include any functional information from stroke patients. Consequently, our previous models were not predictive and could not reproduce the functional connectivity anomalies observed in patients from their structural alterations alone.

The results described below validate the new predictive model by showing that it reproduces FC abnormalities both at the individual and group levels, which resemble the empirical findings reported in the literature. Moreover, the new model has the same degree of accuracy as our previous models, which were fit directly to the patient's functional data. In addition to supporting the model validation, these results show that the model mechanistically captures the relationships between the anatomical structure and the functional activity in the human brain.

## Materials and methods

### Subjects

We utilized data from the Washington University Stroke Cohort dataset,^16^ which is a comprehensive longitudinal study spanning 2 weeks, 3 months and 12 months. It focuses on patients experiencing their first-time, single-lesion stroke, predominantly ischemic (83%) with a minority being haemorrhagic (17%). Our analysis solely concentrated on the data collected at the initial time point, within 1–3 weeks post stroke (mean = 13.4 days, SD = 4.8 days). Additionally, we examined a control group matched for age, consisting of 27 healthy individuals who were assessed twice and 3 months apart. From this cohort, we selected 96 stroke patients (M = 57%, F = 43%) and 27 healthy subjects (M = 59%, F = 41%).

The stroke patients were recruited prospectively from the stroke service at Barnes-Jewish Hospital (BJH) in collaboration with the Washington University Cognitive Rehabilitation Research Group (CRRG). The detailed data collection methodology is outlined in full detail in a prior publication.^16^ The healthy controls were chosen based on the same criteria as outlined previously.^16^ Typically, they were spouses or first-degree relatives of the patients, who were matched in age and education level. Patients underwent neuroimaging assessments to obtain structural and functional features and an extensive (∼2 h) neuropsychological battery.

### Neuroimaging acquisition and preprocessing

An extended outline of the neuroimaging evaluation can be found in a previous publication.^18^ The neuroimaging data were gathered at the Washington University School of Medicine using a Siemens 3T Tim-Trio scanner equipped with a 12-channel head coil. Specifically, the protocol involved acquiring the sagittal T1-weighted MP-RAGE (TR = 1950 ms; TE = 2.26 ms, flip angle = 90°; voxel dimensions = 1.0 × 1.0 × 1.0 mm), as well as a gradient echo EPI (TR = 2000 ms; TE = 2 ms; 32 contiguous slices; 4 × 4 mm in-plane resolution) from each participant. During the scans, subjects were instructed to focus on a small white fixation cross against a black background on a screen located at the back of the magnet bore. Each participant underwent between six and eight resting-state scans each consisting of 128 volumes, totalling to approximately 30 min of scanning time and yielding 896 time points per participant.

Preprocessing of resting-state fMRI data involved the following steps: (i) regression of head motion parameters, signals from the ventricles, CSF and white matter; (ii) global signal temporal filtering retaining frequencies in the 0.009–0.08 Hz band; and (iii) censoring of frames with large head movements, FD = 0.5 mm. The resulting residual time series was mapped onto the cortical and subcortical surfaces of each participant's brain, which was partitioned into 234 regions of interest (200 cortical and 34 subcortical). These regions were selected from the multi-resolution functional connectivity-based cortical parcellations developed by Schaefer *et al.*,^29^ supplemented by additional subcortical and cerebellar parcels from the automated anatomical labelling (AAL) atlas^30^ and a brainstem parcel from the Harvard–Oxford Subcortical atlas (https://fsl.fmrib.ox.ac.uk/fsl/fslwiki/Atlases).

Furthermore, a structural connectome atlas was constructed using a diffusion MRI streamline tractography atlas publicly available based on the high-angular resolution diffusion MRI data obtained from 842 healthy participants in the Human Connectome Project.^28^ This atlas, previously reported (Griffis *et al*., 2019, 2021), was built using high-spatial and angular resolution diffusion MRI data reconstructed in the MNI template space using q-space diffeomorphic reconstruction.^31^ The resulting spin distribution functions were averaged across all 842 subjects to estimate the normal population-level diffusion patterns. Whole-brain deterministic tractography was then conducted on the population-averaged dataset, employing multiple turning angle thresholds to obtain 500 000 population-level streamline trajectories.

The predictive patient model presented in this study was based partly on a group structural representation (i.e. a group healthy structural connectome) and partly on patient-specific structural information (i.e. the patient's lesion). As such, inaccuracies in how well the group healthy connectome corresponds to a patient's pre-stroke structural connectome will affect the model accuracy for that patient. Therefore, it is relevant to clarify that for stroke patients, the inter-individual variability in structure is dominated by the disconnection caused by the lesion, rather than fine-grained individual differences in the pre-stroke connectome.

### Neuropsychological and behavioural assessment

The same subjects (controls and patients) underwent a comprehensive battery of neuropsychological assessments at each time interval. This battery comprised 44 measures distributed across four functional domains: language, motor, attention, and memory (for detailed descriptions of the task measures, see the work of Corbetta *et al.*^16^). Additionally, a perimetric evaluation of visual fields was conducted. Within each domain, individual test data underwent a dimensionality reduction via the principal component analysis following the method outlined by Corbetta *et al.*,^16^ yielding summary domain scores: language, MotorR and MotorL (one score per side of the body), AttentionVF (visuospatial field bias), attention average performance (overall performance and reaction times on the battery's attention tasks),AttentionValDis (ability to reorient attention to unattended stimuli), MemoryV (composite verbal memory score) and MemoryS (composite spatial memory score). Furthermore, patients' behavioural scores were standardized using z-scores in relation to the scores of controls, facilitating the identification of behavioural deficits.

In addition to domain-specific scores, the patients' clinical severity was evaluated using the National Institutes of Health Stroke Scale (NIHSS)^32^ that encompasses 15 subtests addressing the level of consciousness (LOC), gaze and visual field deficits, facial palsy, upper and lower motor deficits, limb ataxia, sensory impairment, inattention, dysarthria and language deficits. The total NIHSS score provided an averaged measure of clinical severity for each patient.

#### Functional connectivity measures

Based on previous work,^17,18^ we defined three measures that are consistently impaired in stroke patients:

1. Intra-hemispheric FC: average pairwise FC between regions of the dorsal attention network (DAN) and the default mode network (DMN)
2. Inter-hemispheric FC: average homotopic inter-hemispheric connectivity within each network
3. Modularity: overall Newman's modularity among cortical networks, a comparison between the number of connections within a module and the number of connections between modules. Here, by ‘module’ we mean what is commonly called ‘module’ or ‘community’ or ‘cluster’ in network theory (i.e. a set of nodes forming a tightly knit subnetwork). In this case, modules correspond to major resting state networks.^33^

#### Lesions

Manual segmentation of each lesion was performed on structural MRI scans and verified by two board-certified neurologists. The categorization of the lesion location (cortico-subcortical, subcortical, white-matter only) was determined using an unsupervised K-means clustering based on the percentage of the total cortical/subcortical grey and white matter masks overlay. For a detailed explanation on how the overlap of each lesion group with grey matter, white matter and subcortical nuclei is calculated, please refer to Corbetta *et al.*^16^

#### Lesion disconnection masks

The Lesion Quantification Toolkit (LQT)^34^ generates a comprehensive set of atlas-derived lesion measures that includes measures of grey matter damage, white matter disconnection and alterations of a higher-order brain network topology. Crucially, the measures are derived from population-scale (e.g. *N* = 842) atlases of grey matter parcel boundaries and white matter connection trajectories established using high-quality resting-state functional MRI and diffusion MRI data.^28^

Taking advantage of the LQT, structural disconnection (SDC) masks were constructed to represent spared connection, quantifying the percentage of streamlines connecting each region pair in the atlas-based structural connectome that remained unaffected by the lesion. Consequently, the multiplication of each SDC with the aforementioned group-average structural connectivity^34^ provides an atlas-based weight for each region pair in each subject. The SDC masks were generated by integrating indirectly derived measures of structural disconnection into the healthy structural connectivity atlas. In the same cohort, we also assessed diffusion tensor imaging (DTI) at 3- and 12-months post-stroke, but these time points were not included in this study.

Given that many stroke lesions primarily affect the white matter or include both a grey and white matter component, the SDC mask offers an accurate depiction of connectome damage. We computed the total amount of disconnection^18^ as a metric of anatomical impairment to assess the validity of the model. For each patient, region-based disconnections were binarized at a 1% disconnection threshold. The total disconnection score for a patient was then determined by summing the number of disconnected regions. Therefore, the disconnection is obtained from each subject SDC, for which the relevance is driven, but not the amount and location or consequences of the lesion, as reported in previous literature.^14,18,34^

### Whole-brain Hopf model parameter estimation

The Hopf model directly simulates the BOLD activity across the entire brain. It comprises interconnected dynamical units representing cortical and subcortical brain regions based on a specified parcellation. The local dynamics of each brain region (node) is described by the normal form of a supercritical Hopf bifurcation, also known as a Stuart–Landau oscillator, which is the canonical model for investigating the transition from a stable equilibrium to a periodic oscillation. Our approach involved employing the Hopf computational model to simulate the whole-brain BOLD activity, capturing the emergent dynamics arising from the interactions among interconnected brain regions, as determined by established anatomical structural connectivity graphs.^35,36^ The structural connectivity matrix (group average SC template) was scaled to a maximum value of 0.2^36^ to explore the range of the G parameter established in the previous work. To calculate the generative effective connectivity (GEC), we optimized the phase of the empirically measured FC in the healthy subject group with the phase of the model FC time series. This optimization was performed by changing the global coupling parameter G (obtaining a value of G = 0.75 as the optimal one), which assesses the influence of SC in the model. A higher G value indicates a greater influence of the system on each node. The model encompasses 234 coupled dynamical units (ROIs or nodes) representing the 200 cortical and 34 subcortical brain regions from the parcellation. Integration with brain network anatomy, as described in the ‘Neuroimaging acquisition and preprocessing’ section, enables the complex interaction between Hopf oscillators to effectively replicate features of brain dynamics observed in fMRI.^35,36^

In complex coordinates, each node j is described by the following equation (for further details, refer to Deco et al.^37^):


(1) $$\frac{dz_{j}}{dt}=z(a_{j}+i\omega_{j}-{\left|z_{j}\right|}^{2})+g\;\overset{N}{\underset{k=1}{\sum }}\;C_{\;jk}\;(z_{k}-\;z_{j})+\;\beta \eta_{j},$$

and


(2) $$z_{j}=p_{j}e^{i\theta }=\;x_{j}+iy_{j}$$

where *α* and *ω* denote the bifurcation parameters and intrinsic frequencies of the system, respectively. This normal form exhibits a supercritical bifurcation at $a_{j}$ = 0 for where we utilized a homogeneous parameter space around the Hopf bifurcation (a = −0.01). Within this model, each node's intrinsic frequency $\omega_{j}$ falls within the 0.04–0.07 Hz range (j = 1, …, *n*). The intrinsic frequencies were derived from the data calculated as the averaged peak frequency of the narrowband BOLD signals of each brain area. The variable G represents a global coupling factor scaling the structural connectivity C_jk_, and η represents a Gaussian noise vector with standard deviation β=0.04. Conceptually, this model extends the Kuramoto model with amplitude variations, thus the choice of coupling ($z_{k}-\;z_{j}$) indicative of a synchronization between two coupled nodes. We incorporated Eq. 2 in Eq. 1 separating the real part in Eq. 3 and the imaginary part in Eq. 4.^36^


(3) $$\frac{dx_{j}}{dt}=(a_{j}-x_{j}^{2}-y_{j}^{2})x_{j}-\omega_{j}y_{j}+G\;\underset{k}{\sum }\;C_{\;jk}(x_{k}-x_{j})+\;\beta \eta_{j}(\tau )$$

(4) $$\frac{dy_{j}}{dt}=(a_{j}-x_{j}^{2}-y_{j}^{2})y_{j}+\omega_{j}x_{j}+G\;\underset{k}{\sum }\;C_{\;jk}(y_{k}-y_{j})+\beta \eta_{j}(\tau )$$

For all cases, we will compute the goodness of fit by the mean error (squared difference) between the upper triangular values of the empirical and simulated FC.

### Generative effective connectivity calculation

The generative effective connectivity (GEC) utilizes differences detected at different times in the signals for the connected pair of brain regions to infer what effects one brain region has on the other.

The analysis of the GEC integrates an indirect measure into the whole-brain model, replacing the existing descriptive metrics of FC and SC. Previous researches have demonstrated the pivotal role of the GEC in elucidating information propagation within structural networks.^38,39^ The methodology for estimating the GEC is extensively elucidated in a prior publication.^37^ In essence, we computed the distance between our model and the empirical grand average phase coherence matrices serving as a measure of system synchronization within the healthy control group. Each structural connection was individually adjusted using a greedy version of the gradient-descent approach. All values were transformed into a mutual information measure to work only positive values for the algorithm (assuming a Gaussian distribution). We derived the healthy simulated functional connectivity $FC^{model}$ from the first N rows and columns of the covariance matrix *K* representing the real part of the dynamics, specifically mirroring the BOLD fMRI signal. Subsequently, we optimized parameter ‘C’, such that the model optimally replicates both the empirically measured covariances $FC^{empirical}$ (i.e. normalized covariance matrix of the functional neuroimaging data) and the empirical time-shifted covariances $FS^{empirical\;}(\tau )$, where τ is the time lag normalized for each pair of regions *i* and *j* by $\sqrt{KS_{\;jk}^{empirical}(0)KS_{\;jk}^{empirical}}(0).$ The optimization process was iteratively performed until full optimization was achieved. The equation of the optimization is as follows (for detailed information, please refer to the relevant citation):


(5) $$C_{\;jk}=C_{\;jk}+\;\varepsilon (FC_{\;jk}^{emp}-FC_{\;jk}^{mod})+\;\varepsilon (FS_{\;jk}^{emp}(\tau )\;-FS_{\;jk}^{mod}(\tau )).\;$$

where *C* is the anatomical connectivity updated with the difference between the grand-averaged phase coherence matrices (empirical: $FC_{\;jk}^{emp}$ and model: $FC_{\;jk}^{mod}$) and the difference between the time-shifted covariance matrices both scaled by a factor ε = 0.001, as extensively reported for optimization through a gradient-descent approach in previous literature.^14,27,40^  $FS_{\;jk}^{mod}(\tau )$ is defined similarly to $FS_{\;jk}^{emp}(\tau )$. After this process, *C* is considered as a GEC matrix. Consequently, the prediction relies on the ongoing estimation of the healthy structural connectivity, which is iteratively updated by optimizing the phase FC in each iteration. In essence, the model underwent multiple runs with recursive updates of GEC until convergence was achieved. The distinction between functional and effective connectivities is crucial here. The FC denotes the statistical dependence between distant neurophysiological activities, while the GEC represents the directional influence one neural system exerts over another, imparting asymmetry to the matrices.^41,42^

### Models

#### Full predictive model

**Figure 1:**
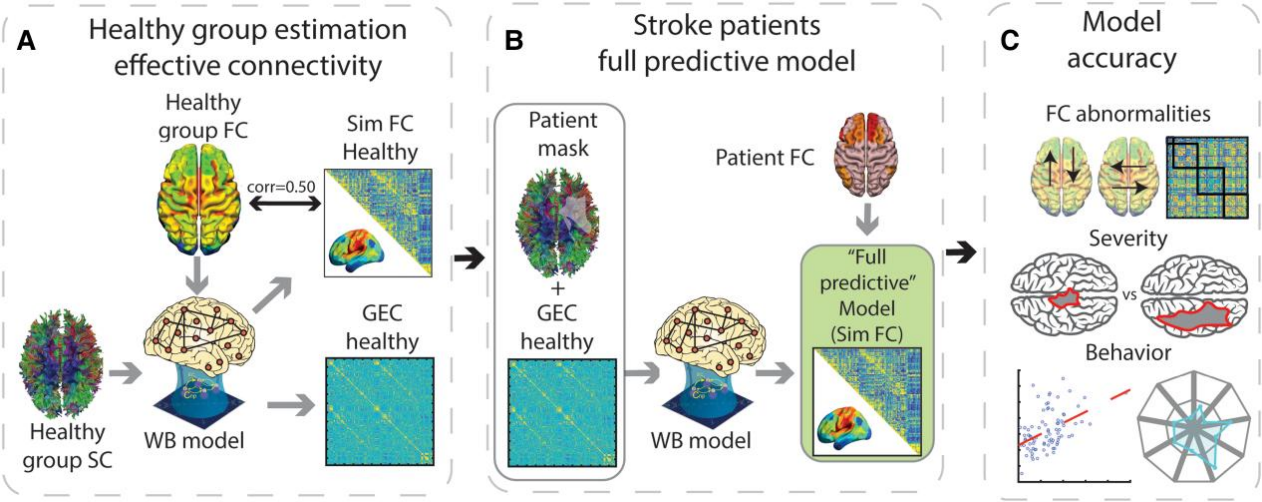
**Pipeline for the predictive model: (A)** Healthy control generative effective connectivity (GEC) was calculated by using the healthy template structural connectivity (SC) with each healthy control fMRI time series. The model was optimized using a whole-brain (WB) model to create an average GEC for the healthy controls. **(B)** The predictive model used each patient's disconnection mask to modify the control GEC and obtain the patient's simulated functional connectivity (FC), referred to in the figure as the full predictive model. **(C)** We determined the accuracy of the healthy model in accounting for the FC matrix of each healthy control subject (Fig. 2A). We also determined the accuracy of the full predictive model in predicting each patient's FC matrix (Fig. 2A), FC-derived measures in each patient that are typically abnormal following a stroke (Fig. 2B), z-scored abnormalities, relative to healthy controls, in the FC matrix of each patient (Fig. 2C) and each patient's behavioural deficits (Fig. 2D, upper panel) . We also investigated the determinants of the model accuracy by examining whether the accuracy of a patient's simulated FC matrix covaried with the severity of their lesion-induced structural damage (Fig. 3A), the magnitude of FC-based and graph-related functional measures that are typically abnormal following a stroke (Fig. 3B and C) and the magnitude of the patient's behavioural deficits (Fig. 3D).

We calculated a predictive model to capture the dynamical effects of stroke lesions 2 weeks after onset. First, the average BOLD time series of each region of interest was Hilbert-transformed to yield the phase evolution of the regional signals, as reported in previous literature as a proxy for brain dynamics similarity.^14,37^ Next, we estimated the optimal value of global coupling for which the modelled Hilbert phases were most similar to the empirical data in the healthy control group (G = 0.75). We then computed the GEC (see previous section) on the same group (Fig. 1A). Lastly, we added the information of the SDC mask of each patient to the existing GEC to simulate the fMRI BOLD data of the corresponding patient (Fig. 1B). Previous models included both structural and functional information from each stroke patient, making them ‘non-predictive’ models. In contrast, the ‘full predictive model’ used to generate the simulated time series contained structural information from the patient, but not their functional information, making it a predictive model.

For each patient, the simulated fMRI BOLD time series for each parcel pair were then correlated to construct the patient's simulated FC matrix. To isolate the degree to which the patient's FC between two parcels was abnormal relative to the healthy controls, the empirical and simulated FC matrices for each patient were z-scored with respect to the healthy controls' empirical and simulated FC matrices to create the patient's empirical and simulated z-scored FC abnormality matrices. Specifically, the healthy group-mean FC for a parcel pair was subtracted from the patient's FC for that parcel pair. This difference score was then divided by the standard deviation of the healthy group FC for that parcel pair.

In addition, we separately averaged the homotopic interhemispheric FC and DAN–DMN entries from a patient's z-scored FC abnormality matrix to create averaged abnormality scores for these two classes of FC, which are typically abnormal in patients.

#### Predictive comparative models

Three different predictive models were calculated to compare their performances with the full predictive model:

- Predictive model without mask: To assess the effect of incorporating a disconnection mask (lesion information) in the predictive model, this model simply consisted of the healthy group model without any disconnection mask.
- Surrogate mask model: As the effect of the disconnection mask could simply reflect the overall magnitude of disconnection, we computed predictive models in which each patient received the disconnection mask of another patient. As the lesion severity averages out across patients, but the pattern/location of the lesion is different, comparisons of the full predictive model versus the surrogate mask model indicate how strongly the accuracy of a predictive model depends on incorporating the specific features of a patient's lesion. Therefore, the model without mask and the surrogate mask models serve as predictive controls for the full predictive model.
- G-DSC model: To estimate the contribution of the GEC parameters to the accuracy of the full predictive model, we constructed a healthy model that was fit using only the G parameter, and not the GEC parameters. Therefore, the connections between parcels in this healthy model were based on a healthy structural connectome^28^ that was not modified via the GEC parameters. As for the full predictive model, the healthy model was then lesioned separately for each patient using the patient's structural disconnection matrix.

#### ‘Non-predictive and patient-specific’ model

A non-predictive patient-specific model was calculated for comparison with the full predictive model. The patient-specific model used each patient's own functional information (i.e. their BOLD time series) and their structural disconnection matrix to estimate the model parameters, making the model non-predictive. By including both anatomical and functional information, the patient-specific model could be used to predict the effect of perturbations in the system, such as stimulation.

### Assessment of the model accuracy

To complement the typical metrics (see section ‘Functional connectivity measures’) from the literature that assesses the functional brain dynamics after stroke,^16,17,23^ we have included the following additional metrics of model accuracy:

#### Behaviour impairment prediction

We explored how well subjects’ behavioural scores were predicted by their empirical and simulated FC. We calculated two partial least-squares regression (PLSR) models using the empirical and simulated FC matrices as predictors. As a control, we included a third model based solely on anatomical information (SDC matrix), as reported in previous literature.^17,18,43^

PLSR is a multivariate regression technique^44^ that is closely related to principal component regression.^45^ Both approaches are especially useful for situations where there are more variables than observations and/or when there is a high collinearity among the predictor variables. Nevertheless, PLSR has important advantages that are primarily caused by the differences in the criteria used for the predictor matrix decomposition.^18^ Detailed descriptions of theory and algorithms behind the PLSR approach are explained in previous literature.^46^

#### Global efficiency

Global efficiency was determined by computing the average inverse shortest path length.^47^ Unlike path length, global efficiency can be calculated, even on disconnected networks, because paths between disconnected nodes are defined to possess infinite length and, correspondingly, zero efficiency. Hence, it proves to be an optimal metric for analysing stroke data. While path length is predominantly affected by long paths, global efficiency is primarily influenced by short paths. Several authors have claimed that this may elevate global efficiency to a superior metric of integration.^48,49^ Global efficiency is calculated as follows, following the methods outlined in previous studies:^47,49^


(6) $$E=\;\frac{1}{n}\;\;\underset{i\in N}{\sum }\frac{\sum j\in N,j\neq i\;d_{ij}}{n-1}$$

where *N* is the set of all nodes in the network; *n* is the number of nodes; and ($d_{ij}$) is the shortest path length between nodes i and j.

#### FC entropy

FC entropy is an information theoretical metric that measures the richness of functional connections and may, therefore, be a relevant biomarker for many disorders.^25,26,50^

Previous studies have reported abnormal FC entropy values when comparing healthy controls with stroke patients.^23,25^ However, these models use generic anatomical connectomes based on group averages instead of personalized structural connectivity. Although the current study used an atlas-based structural connectome for modelling the healthy control subjects, this connectome was separately modified for each patient based on their lesion.

Entropy is calculated as follows:^26^


(7) $$H=\;-\overset{m}{\underset{i=1}{\sum }}\;p_{i}\log\;p_{i}/log_{m}$$

where, *m* represents the number of bins employed in constructing the probability distribution function of the upper triangular elements of |FC|. The normalization factor in the denominator is the entropy of a uniform distribution, guaranteeing that H falls within the normalized between 0 and 1.

#### Average degree

Average degree is the measure of the overall network connectivity that provides information about network segregation and integration.^49^

Average degree is calculated as follows:^26^


(8) $$K=\;\frac{\sum_{v}\;k_{v}}{N}$$

where, *N* is the number of nodes, and $k_{v}$ is the degree of node *v* defined above.

### Statistical analysis

We performed paired/unpaired parametrical statistical analyses (*t*-test/ANOVA, based on the group number) depending on whether the comparison was performed for the same patient or between subjects. Statistical significance was assessed with a threshold of *P* = 0.05. All *post hoc* comparisons were Bonferroni-corrected for the number of comparisons taking place.

## Results

To infer the dynamical effects of stroke lesions 2 weeks after onset, we used a computational model based on coupled Stuart–Landau oscillators (Fig. 1A). The model contains a global scale factor also referred to as the G coupling value that determines the influence of SC in the model. It also contains GEC parameters that capture directional interactions between regions. Both parameter types are optimized to improve the model fit (i.e. the similarity of empirical and model FC). In the current study, we use only the functional data of the healthy control dataset to optimize these parameters at the group level. Performing an exhaustive exploration of the homogeneous parameter space (a, G) around the Hopf bifurcation (a = −0.01), we found G = 0.75 as the optimal value of G for which the modelled FC of the Hilbert phases were most similar to those observed in the empirical data. Initializing the GEC to be equal to the SC, we iteratively adjusted its values to improve the similarity between the model and the empirical FC at the group level. Supplementary Fig. 1 shows the role of the structural connections between parcels by assessing the difference between the empirical and simulated FC. We then added information about the structural damage caused by a patient's stroke to the healthy group GEC by using a structural disconnection mask to create a predictive model that generated a simulated version of each patient's FC matrix (Fig. 1B). Therefore, the full predictive model reproduces the functional consequences of stroke lesions in individual patients by exploiting the patient's structural disconnection matrix. Importantly, in contrast to previously reported models, the full predictive model uses the functional data of only healthy control subjects to predict a patient's FC.

From the obtained simulations, several metrics were calculated to assess how well the predictive model captured the functional effects of stroke. For this purpose, among other results, we considered the main FC-based metrics that are known to give abnormal values in stroke patients (mean intra-hemispheric FC between the DAN and DMN, mean homotopic interhemispheric FC and modularity level). Similarly, we determined how well the predictive model captured patients' behavioural deficits. To better understand the determinants of model accuracy, we also examined whether the accuracy of the predictive model for a patient covaried with the magnitude of the structural and FC- and graph-based measures that index the severity of the patient's stroke, such as total structural disconnection, modularity and global efficiency (Fig. 1C).

### Model outcomes and their relationship with stroke effects

**Figure 2:**
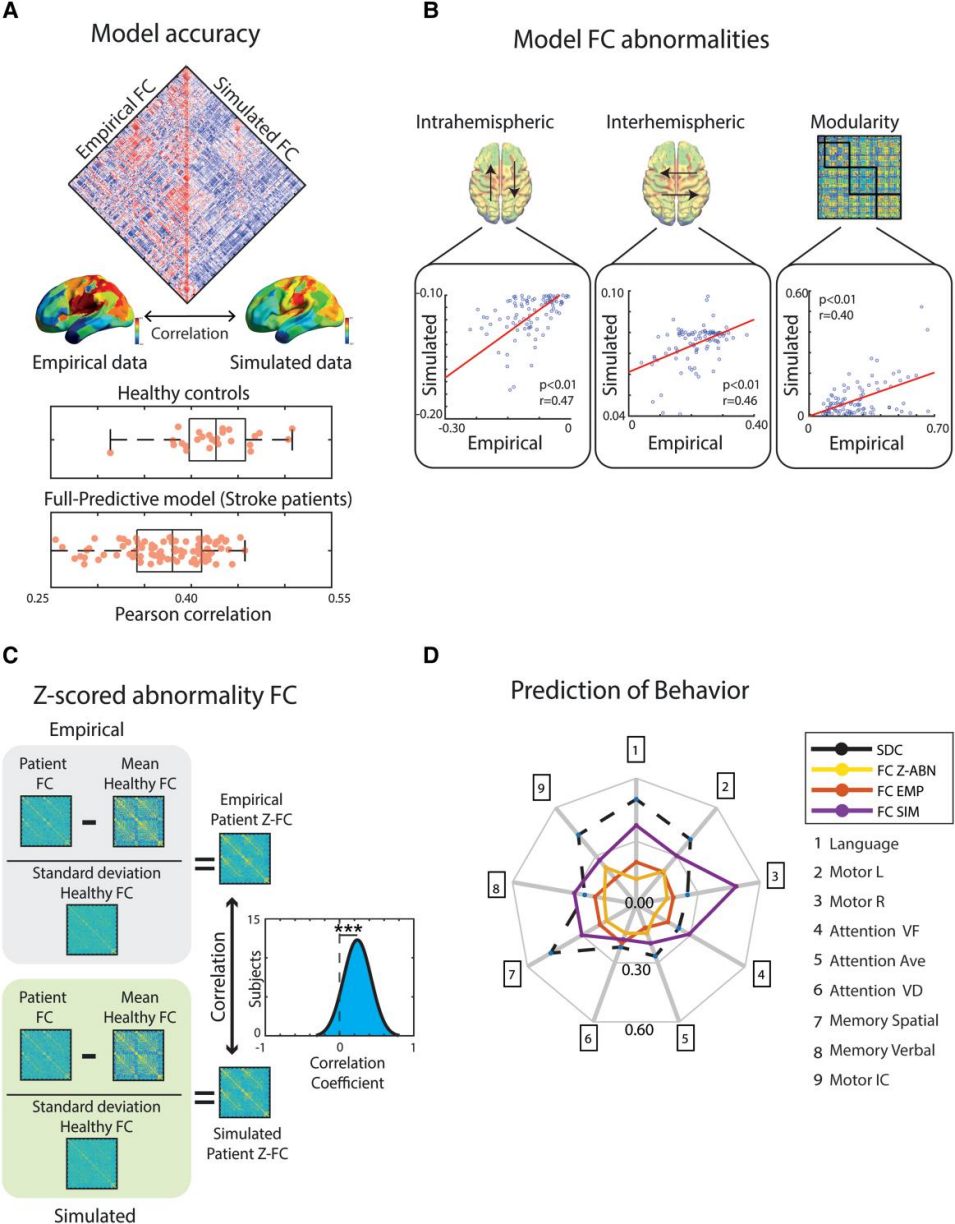
**Model prediction of patient FC and behaviour. (A)** The Pearson correlation between the empirical and simulated FC matrices of each subject (27 healthy controls and 96 stroke patients) was computed to assess the model accuracy (healthy controls: r = 0.43; stroke patients: r = 0.37). **(B)** FC-based measures typically abnormal in stroke patients were calculated from the empirical and simulated FC matrices from the full predictive model to compare their similarities (*N* = 96). **(C)** Distribution across patients of the correlation between the empirical and simulated z-scored FC abnormality matrices for each patient from the full predictive model (*N* = 96). **(D)** Separate partial least squares regression (PLSR) analyses were conducted using the empirical (FC EMP), z-scored (FC Z-ABN) and simulated FC (FCSIM) matrices from the full predictive model and the structural disconnection matrix (SDC) as regressors to predict each domain of behavioural impairment (language; motor left; motor right; attention-visuospatial field bias; attention-average performance; attention-orientation to unattended stimuli; memory spatial; memory verbal; and motor independent component) (*N* = 96).

We first determined how well the model accounted for each subject's empirical FC matrix (healthy controls and stroke patients) by computing the Pearson correlation between the model-generated FC matrix and their empirical FC matrix. This analysis directly compares the similarity of the simulated FC edges to the empirical FC edges, as extensively reported in the literature.^22,37,51^ Fig. 2A shows the distribution of the Pearson correlation coefficients over the sample, indicating that both the healthy and full predictive models generated simulated FC matrices that showed a moderate level of accuracy (healthy group mean, r = 0.43, std = 0.05; patient group mean, r = 0.37, std = 0.05; Fig. 2A).

To assess whether the full predictive model accurately specifically predicted the abnormalities in a patient's FC that resulted from their stroke, we correlated each patient's empirical and simulated z-scored FC abnormality matrices. We performed this analysis by normalizing the matrix with the healthy control group (Healthy FC: *mean* = 0.12, *std* = 0.02; Z-Empirical: *mean* = 0.046, *std* = 0.01; Z-Simulated: *mean* = 0.039, *std* = 0.01). Figure 2C shows that the mean correlation across patients (*r* = 0.24, *std* = 0.18) was significantly positive *(t*(95)= 12.65, *P* < 0.01), indicating that patient abnormalities in FC were significantly predicted.

Next, we analysed how well the model predicted specific FC-based measures that are typically abnormal in stroke patients: (1) a decrease of negative intra-hemispheric FC between regions of the DAN and DMN; (2) a decrease of inter-hemispheric homotopic FC; and (3) a decrease of modularity. Figure 2B shows that the empirical and simulated values for these three signatures of stroke were significantly correlated across patients (intra-hemispheric: *r* = 0.47, *P* < 0.01, MSE = 0.01; inter-hemispheric: *r* = 0.46, *P* < 0.01, MSE = 0.008; modularity: *r* = 0.40, *P* = 0.03, MSE = 0.07). When exploring the levels of intra-hemispheric connectivity, we focused on the connectivity between DAN and DMN because this comparison has been extensively used in the literature.^14,17,18,20,21,52,53^ As this metric is not performed globally, but in specific regions, we complemented it with other whole-brain metrics, such as inter-hemispheric homotopic FC level and modularity. We also included a diverse set of functional dynamic metrics that capture the global impact of stroke lesions (see section ‘Assessment of model accuracy’).

Previous studies have shown that whole-brain GEC models preserve the same three FC-based measures, especially when they include structural disconnection information, revealing the key importance of incorporating this information into the models.^14^ In addition, for each patient, we separately averaged the entries in their z-scored FC abnormality matrix for interhemispheric homotopic FC and DAN-DMN FC to assess whether the model specifically predicted patient abnormalities in these two FC measures. The results shown in Supplementary Fig. 2 indicate that abnormalities in both FC measures were significantly predicted. Overall, the results indicate that the full predictive model reproduced, to some extent, patient FC matrices, patient-specific abnormalities in the FC matrix, summary FC-based measures that are typically abnormal following a stroke and patient-specific abnormalities in those measures.

Finally, we used partial least squares regression (see Methods) to separately assess how well the simulated FC matrices, empirical FC matrix, z-scored FC abnormality matrix and SDC matrix predicted the patients’ behavioural scores. Although the simulated FC matrix was modestly predictive across behavioural domains, the model-independent SDC matrix showed a nominally better performance, except for the domains for Attention VF, Attention Ave and Memory Verbal (Fig. 2D). The empirical FC matrix showed a lower performance than the z-scored and simulated FC matrices across all domains possibly because the predictive model that generated a patient's simulated FC matrix explicitly incorporated their structural disconnection matrix. Overall, these results indicate that although the predictive model partly accounted for behavioural abnormalities, the model did not generally perform better than a purely structural measure.

### Model accuracy in relation to structural damage and global metrics

Next, we investigated whether structural and functional features influenced how accurately the model accounts for the data from individual patients. Specifically, we examined whether the accuracy of the model's simulation of a patient’s FC matrix, as indexed by the correlation between the patient's simulated and empirical FC matrices, covaried with the structural damage from the patient's own lesion, the values of the patient's graph-based functional metrics and the magnitude of FC abnormality metrics that are canonically affected in stroke.

**Figure 3:**
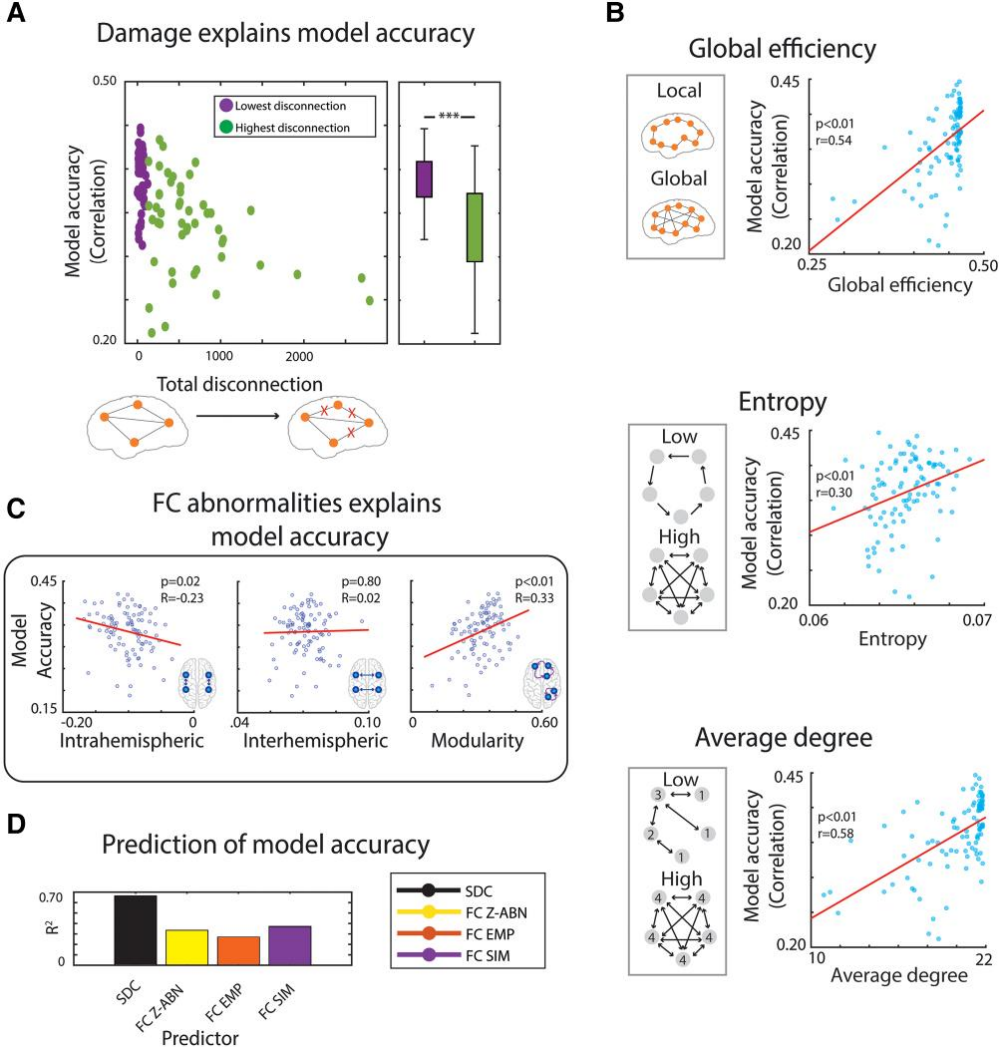
**Structural and functional determinants of model accuracy for individual patients. (A)** Subjects with lower levels of disconnection exhibited a higher correlation between the empirical and simulated FC matrices, indicating a better model performance for patients with less severe lesions (*t*(94) = 5.82, *P* < 0.01; *N* = 96). **(B)** Higher global efficiency, entropy and average degree associated with a higher model accuracy. **(C)** Model accuracy for the FC-based measures typically abnormal in stroke patients assessed for the presented model. The accuracy of the full predictive model was significantly associated with the magnitude of the intrahemispheric FC and modularity, but not interhemispheric FC. **(D)** Model accuracy predicted by each type of regressor (structural disconnection—‘SDC’ matrix, z-scored FC abnormality matrix and FC empirical and FC simulated matrices) in a PLSR analysis of model accuracy (*N* = 96).

We found that higher model accuracy was associated with lower values of total structural disconnection (R: −0.58, *P* < 0.01, MSE: 0.04), which served as a measure of overall lesion damage (Fig. 3A, left panel). When splitting the sample in half by using the median value of total structural disconnection, patients with greater total disconnection showed a significantly lower model accuracy (*t*(94) = 5.82, *P* < 0.01) (Fig. 3A, right panel). A similar analysis using lesion volume (number of damaged voxels) as an alternative metric yielded similar results (Supplementary Fig. 3; an example of a patient lesion and the corresponding asymmetric effective connectivity matrix is shown in Supplementary Fig. 4).

The dependence of the model accuracy on lesion severity was consistent with its dependence on the magnitude of graph-based metrics that are typically abnormal following a stroke.^26^ We found significant positive correlations between model accuracy and global efficiency (*r* = 0.54, *P* < 0.01, MSE = 0.04; Fig. 3B-Top), entropy (*r* = 0.30, *P* < 0.01, MSE = 0.05; Fig. 3B-Middle) and average degree (*r* = 0.58, *P* < 0.01, MSE = 0.04; Fig. 3B-Bottom). In other words, model accuracy was higher when the lesion produced weaker network function abnormalities.

Furthermore, the full predictive whole brain model generated FC matrices whose correlation with empirical FC matrices (i.e. model accuracy) was significantly related to the magnitudes of intra-hemispheric FC (*R* = −0.23, *P* = 0.02, MSE = 0.04) and FC modularity (*R* = 0.33, *P* < 0.01, MSE = 0.04), as seen in Fig. 3C. The sign of the relationship was consistent with the conclusion from Fig. 3A and B that the model more poorly predicted the functional measures of patients that had more abnormal structural or functional measures. However, model accuracy was not correlated with the magnitude of inter-hemispheric FC, which is typically lower in stroke patients than controls.

Finally, separate PLSR analyses showed that model accuracy for a patient was well predicted by the full SDC matrix, next by the simulated FC matrix and z-scored FC matrix and least by the empirical FC matrix (SDC: R^2^ = 0.66, *P* < 0.01, Sim-FC: R^2^: 0.37, *P* < 0.01, Z-scored Abnormality FC: R^2^: 0.35, *P* < 0.01, and Emp-FC: R^2^: 0.27, *P* < 0.01) (Fig. 3D).

Overall, these results indicate that the model's ability to accurately reproduce a patient's FC matrix decreased as the patient's structural measures and, to a lesser extent, functional measures showed larger departures from those for healthy controls.

### Model comparisons

**Figure 4:**
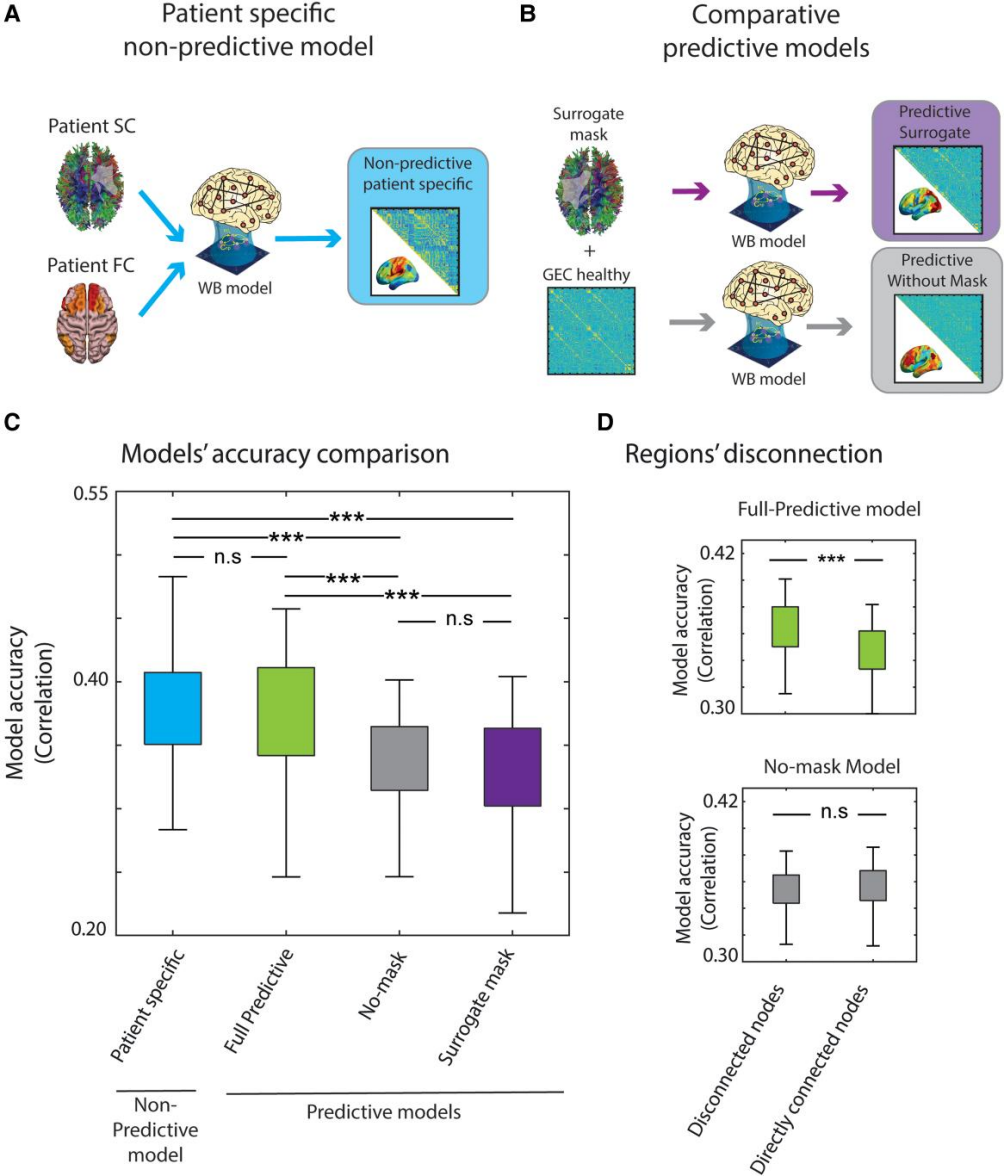
**Model comparisons. (A)** We calculated the non-predictive patient specific whole-brain (WB) model using both the anatomical and functional data of each patient. This model was not predictive because it was fit to the patient’s functional data. **(B)** We calculated two comparative predictive models to compare with the full predictive model. The predictive no-mask model was built using only the healthy generative effective connectivity (GEC), while the predictive surrogate mask model was calculated by modifying the healthy GEC via the disconnection mask of a different patient. **(C)** The similarity between the empirical and simulated FC matrices was assessed for each model. A within-subject ANOVA indicated that the non-predictive patient specific model and full predictive model showed similar levels of performance that exceeded that of the surrogate and no-mask models (*F*(3285) = 14.84, *P* < 0.01; *N* = 96). **(D)** A two-way ANOVA indicated that the comparison of the model performances when dividing by disconnected nodes and directly connected nodes showed a significant interaction between model and node connection (*F*(1, 190) = 67.96, *P* < 0.01; *N* = 96).

Having established the accuracy with which the full predictive model reproduced measures that are typically abnormal in stroke patients (Fig. 2B and C), we compared its performance with a patient-specific non-predictive model reported in a previous study^14^ that used both anatomical and functional information to simulate a patient's time series (referred to as the non-predictive patient specific model) (Fig. 4A).

Secondly, two comparative predictive models were chosen to assess how much model accuracy depended on the patient's disconnection mask that was incorporated in the full predictive model. The model without mask allowed us to assess the accuracy gain obtained by incorporating the patient's lesion information with respect to not incorporating any lesion information (i.e. using the unmodified healthy group model for all patients). The surrogate mask model allowed us to assess the accuracy gain obtained by specifically incorporating the patient's lesion information with respect to using a lesion from a different patient (Fig. 4B).

We compared the performances of all models by computing the correlation of the simulated and empirical FC matrices. The full predictive model showed a roughly equivalent accuracy to the non-predictive patient specific model,^14^ while the model with surrogate mask and that without mask showed lower accuracies (Fig. 4C).

A within-subject ANOVA indicated that the main effect of the model type (non-predictive, full predictive, no mask and surrogate mask) on accuracy was significant (*F*(*3*285) = 14.84, *P* < 0.01). *Post-hoc* tests indicated that the patient-specific and full predictive models did not significantly differ in accuracy (*P* < 0.36), but were significantly more accurate than both the surrogate mask and no mask models (*P* < 0.01 in all cases).

To assess the effects of the GEC parameters on model accuracy, a healthy model was fit using only the G parameter, not the GEC parameters, and was then lesioned as in the full predictive model to generate predictions for the patients. The results shown in Supplementary Fig. 10 indicate that model accuracy was significantly higher for the full predictive model than for the G-DSC model.

The accuracy of the full predictive model depends on the integration of functional and structural information; hence, we assessed this relationship in more detail. Specifically, model accuracy was computed as a function of whether two parcels were directly or indirectly connected (Fig. 4D). A two-way ANOVA was performed to analyse the effect of the model type and the node connection on the model accuracy. The two-way ANOVA revealed a statistically significant interaction (F(1, 190) = 67.96, *P* < 0.01). The full predictive model showed a significantly greater accuracy for indirectly connected parcels compared to directly connected parcels (*P* < 0.01), but showed no difference for the no mask model (*P > 0*.05).

The model accuracy relative to the accuracy of the healthy control group model is shown in Supplementary Fig. 5. The influence of the global coupling parameter (GC) is presented in Supplementary Fig. 6. The relation between the accuracy of each model and the magnitude of the FC measures typically abnormal in stroke patients is presented in Supplementary Fig. 7A, while the association between the different FC abnormalities is displayed in Supplementary Fig. 7B. Comparisons of the dynamical metrics^22,51^ between the models are presented in Supplementary Fig. 8 (see figure caption for metric explanation).

Overall, the results show the efficacy of the full predictive model, which does not use a patient's functional BOLD data, allowing its predictions to be generalized to new patient datasets and opening the door for predicting the expected effects of a simulated lesion or external stimulation of a patient's brain.

## Discussion

The results show that functional connectivity in patients could be predicted by a whole-brain computational model strictly from the structural disconnection caused by a patient's lesion, suggesting that the model mechanistically captured to some degree the relationship between anatomical structure and functional activity. Moreover, the model significantly predicted abnormalities in patient FC with respect to the FC of the healthy control group. Although the model also predicted the behavioural abnormalities of patients, prediction was no better than that obtained using a purely structural measure, namely, the structural disconnection matrix. While previous work has examined how well computational models can reproduce FC when the model parameters are directly fit using functional and structural data from healthy controls or stroke patients,^14,52^ the current study moves fundamentally beyond such work by determining whether these models can actually predict the effects of a stroke based solely on the structural information associated with a patient's lesion.^26^

### Validating the full predictive model's integration of structure and function

The Introduction noted that the large number of free parameters in whole-brain models emphasizes the need for strong validation procedures, such as the out-of-population approach taken here. Both the facts that the full predictive model significantly predicted patient abnormalities and that the accuracy of the predictive model was essentially equivalent to the accuracy obtained by fitting a non-predictive model directly to the patient's functional and structural data, despite the absence of free parameters in the full predictive model, provide support for model validity. Moreover, because the out-of-population prediction was applied to a patient population with a different structural connectome than the training population, the variation of model accuracy with lesion severity, connection type (direct, indirect) and lesion mask type (the patient's own mask, a different patient's mask, no mask) provided insights into the conditions under which the full predictive model best integrated anatomical structure with functional activity.

We first fit a model to data from age- and education-matched healthy controls based on a healthy structural connectome and the healthy controls' functional imaging data. For each patient, we then determined how the patient's lesion has modified the healthy structural connectome and made corresponding changes to the structural connectivity parameters in the healthy model (the GEC parameters) without additional model fitting or recourse to the patient's functional data. Finally, the modified healthy model specific to the patient (i.e. the full predictive model) generated the patient's predicted FC, which was compared against the empirically measured FC. Specifically, model accuracy was evaluated by assessing the correlation between the patient's empirical and predicted FC matrices. The FC matrices specify the functional interactions between each pair of brain regions; therefore, the predicted matrices potentially provide information concerning which functional connections are particularly vulnerable in the patient, a possibility also raised by the prediction of the patients' z-scored FC abnormality matrices.

Interestingly, the accuracy of the full predictive model was essentially equivalent to the accuracy obtained by fitting the model directly to the patient’s functional and structural data. Moreover, the accuracy of the full predictive model depended on incorporating the structural disconnection specific to that patient's lesion, as shown by the significantly poorer performance obtained by substituting the structural disconnection for a different patient. This is derived from the fact that a higher level of structural disconnection (more severe lesions) deviates more from the trained sample (consisted in healthy controls). Both results provide additional support for how the full predictive model integrated structure and function.

However, the accuracy for predicting a patient's FC matrix tended to be less more because the patient's structural connectome and functional measures differed from the structural connectome and functional measures of healthy control subjects. Specifically, accuracy decreased with the magnitude of the total structural disconnection caused by the lesion. Similarly, the measures of modularity and intra-hemispheric FC and graph-based measures that are typically abnormal following a stroke tended to be more poorly predicted, and the more they differed from healthy control values (surprisingly, a similar relationship was not observed for inter-hemispheric FC, an important signature of stroke-induced dysfunction).

One interpretation is that the model tended to better predict patients that were more similar to healthy controls because it was initially based on a model computed from healthy control data.

This interpretation suggests a limitation on how well the full predictive model integrated anatomical structure and functional activity, an interpretation also suggested by the difference in model accuracy for node pairs that were directly versus indirectly connected in the healthy structural connectome.

Alternatively, similar relationships may also be present for the non-predictive patient-specific model (i.e. the dependence of model accuracy on structural and functional measures may not be related to prediction per se or be a unique feature of the full predictive model). The results in Supplementary Figs 7A and 9 provide some support for this alternative, but those results do not rule out the first explanation as a contributing factor.

Finally, as a further alternative, it can be considered that the larger the amount of damage occurring in the brain, the more frequent the functional effects appear in secondary and tertiary connections, which are not well estimated in the healthy controls (given that healthy control FC does not change its weights). This reasoning would explain why indirect FC measures are not significantly predictive of behaviour, while direct FC measures are, especially for cognitive deficits that involve multi-network pattern abnormalities.^17,20^

### Relation to previous work

The previous computational work based on concepts from statistical mechanics has shown that resting-state organization conforms to a state of ‘criticality’ that promotes responsiveness to external stimulation (i.e. resting state organization facilitates task-based processing).^13,54–57^ The rich body of empirical work on resting-state organization has facilitated an important testing ground for evaluating computational whole-brain models. In these models, neural modules or elements are connected by ‘structural’ links that mirror the empirical structural connectivity of the human brain as assessed using diffusion-based MRI,^27,54,55,58^ resulting in resting-state dynamics that respect critically. Initial applications of whole-brain computational models to stroke populations^23,25^ used the biophysically-based model of Deco *et al*.,^55^ which involves a mean field approximation of populations of spiking neurons with realistic NMDA, AMPA, and GABA synaptic dynamics. However, the authors subsequently developed the mesoscopic Hopf model^37^ used in the current study, which provides a better fit to healthy control data and runs two orders of magnitude faster, allowing the use of higher-resolution functional parcellations that likely increase the model accuracy.

The whole-brain computational models presented in recent studies that involved stroke patients included a global coupling parameter and GEC parameters that encoded directional interactions between nodes that had direct structural connections.^14,52^ The resulting generative effective structural connectivity weights allowed a better fit between the empirical and modelled FC than that achieved by models that only varied the global coupling parameter. In both papers, however, the model was fit directly to a patient's functional data and, therefore, was not a predictive model.

Given the moderate model accuracy and the decrease in the model accuracy as the damage from a stroke increases, we acknowledge a room for improvement in future work, which could include the application of the model to other focal and non-focal pathologies that damage the structural connectome. Although the current study focused on predicting FC in sub-acute stroke patients, future studies could examine whether changes in structural connectivity during recovery also produce predicted changes in FC. The dataset consisted mostly of ischemic patients; hence, model predictions will need to be tested in haemorrhagic stroke patients before concluding that the model applies more generally to stroke. Following this line, the presented model was trained with a healthy group population. Due to the heterogeneity of strokes, a bigger dataset would be needed to train the model directly with patient data. Specifically, a large amount of data would be needed to represent all stroke subgroups (different location, sizes, etc.) such that the model could be appropriately trained. Future studies could benefit from open-source datasets to achieve this goal.

### Limitations

The mesoscopic Hopf model^37^ includes global coupling and GEC parameters that affect the connectivity between nodes of the model and bifurcation parameters that affect the node dynamics. The influence of the GEC parameters is depicted in Supplementary Fig. 10. Specifically, the bifurcation parameter for a node governs the transition between noise-dominated and oscillatory behaviours. The current work assumed that strokes do not affect the bifurcation parameters/nodes, only the connections between nodes; however, prior studies indicated that delta waves are prominent in the perilesional tissue and propagate to directly connected regions.^59,60^ Therefore, the nodes for perilesional/partly damaged parcels and perhaps directly connected parcels may have abnormal bifurcation parameters. Evaluating this possibility is beyond the scope of this work, but is currently in progress. On the positive side, properly accounting for abnormal bifurcation parameters/nodes may improve the model accuracy. On the negative side, it is unclear how node abnormality might be incorporated into a fully predictive model.

Although the GEC parameters encode directed influences between parcels, only undirected influences were assessed in the data via Pearson correlation and used to evaluate the model accuracy. Moreover, it is important to consider that stroke patients often exhibit an older demographic profile compared to the typical populations, upon which the assumptions of neurovascular coupling and conventional analysis pipelines are based.^61^ Additionally, it is essential to interpret the presented results with the awareness that the BOLD signal does not directly measure the neuronal activity. Therefore, changes in network dynamics across different time points could reflect alterations in observable BOLD fluctuations, rather than explicit modifications in neuronal dynamics. Finally, the presented dataset consisted mostly of single-lesion stroke patients. Future studies could evaluate the model accuracy for patients with multiple lesions.

## Conclusion

The current study shows that the effect of stroke-induced perturbations in structural connectivity on functional dynamics can be partly captured by a fully predictive whole-brain computational model, thereby demonstrating how out-of-population analyses can be used to validate whole-brain computational models. Adding lesion information to a model trained on healthy functional data is beneficial for reproducing functional anomalies in patients. Although the prediction accuracy worsens for patients showing greater structural damage and functional deficits, the predictive model can still provide unique insights into how strokes disrupt resting brain organization.

## Supplementary Material

fcae237_Supplementary_Data

## Data Availability

Data could be shared upon request. Simulated data and the corresponding scripts for the presented analysis can be obtained at: https://github.com/SebastianIdesis/Predictive_model-2023-.
