# Supplementary material for: Generative whole-brain dynamics models from healthy subjects predict functional alterations in stroke at the level of individual patients
Source: Brain Commun. 2024 Jul 13;6(4):fcae237. doi: 10.1093/braincomms/fcae237 (PMC11285191; doi:10.1093/braincomms/fcae237)
Supplement: fcae237_Supplementary_Data [file Original_submission_decision_letter.pdf › Original_submission_decision_letter.pdf]

From: brcoms.editorialoffice@oup.com
To:
CC:
BCC:
Subject: Brain Communications - Decision on Manuscript ID BRAINCOM-2024-030
Body: 02-Apr-2024

Dear Dr. Idesis,

Thank you for your recent submission BRAINCOM-2024-030 entitled "Generative whole-brain dynamics models from healthy subjects predict functional alterations in stroke at the level of individual patients." to Brain Communications.

Your manuscript has been reviewed, and while the reviewers and editorial team appreciate many aspects of the paper, they raise some concerns that you will need to address in order for the paper to be considered for publication.

If you can address these concerns, I invite you to submit a revised manuscript and a response to each of the reviewers' comments. The reviewer(s)' comments are included at the bottom of this message.

In addition to the reviewers' comments, please also address our Scientific Editor's comments added below to comply with our requirements for presentation of scientific data at Brain Communications.

Please format your revised manuscript according to our Instructions to Authors
https://academic.oup.com/braincomms/pages/General_Instructions
A summary of key points is attached to help with this. Please note that there can be substantial delays to publication if you don't resubmit in line with our requirements.

To revise your manuscript, click the link below or log into https://mc.manuscriptcentral.com/braincom and enter your Author Centre, where you will find your manuscript title listed under "Manuscripts with Decisions." Under "Actions," click on "Create a Revision." Your manuscript number will be appended to denote a revision.

*** PLEASE NOTE: This is a two-step process. After clicking on the link, you will be directed to a webpage to confirm. ***

https://mc.manuscriptcentral.com/braincom?URL_MASK=1e34e06f3dee45719cdd6893470f2890

Please upload a revised manuscript with changes tracked with track changes or in coloured text.

When submitting your revised manuscript, you will be asked to respond to the reviewer(s)

comments. Please be as specific as possible in your response to all of the comments.

The editorial team at Brain Communications aims to increase transparency in scientific publishing and to demonstrate the value that peer review brings to the scientific process. Please therefore consider whether you are willing to have reviewer(s)' comments on your paper and your response published as supplementary data with the paper if it is accepted. This is entirely optional and will only be done if both the authors and reviewers agree. While submitting your revision there will be an opportunity to review or update the answer you gave previously, if necessary.

IMPORTANT: Your original files are available to you when you upload your revised manuscript. Please delete any redundant files before completing the submission.

Because we are trying to facilitate timely publication of manuscripts submitted to Brain Communications, we would appreciate your revised manuscript as soon as possible. If it is not possible for you to resubmit within 3 months, the option to submit a revision will expire, and we would then consider your revised paper as a new submission. However, if you need a little more time, please get in touch and we can extend this deadline for you.

Once again, thank you for submitting your manuscript to Brain Communications and I look forward to receiving your revision.

Best wishes

Dr. Grega Repovs
Associate Editor, Brain Communications

Professor Tara Spires-Jones
Founding Editor, Brain Communications

Reviewer(s)' Comments to Author:

Reviewer: 1

Comments to the Author
The current manuscript investigates the ability of a computational model to simulate the effect of stroke on fMRI functional connectivity from lesion maps, based on normative data. I think the research question is interesting, and has broad interest in the emerging field of "normative connectivity" modelling that is being used in stroke, as well as other disorders, like OCD. The authors show that their model is capable of reproducing several key phenomena that are observed in empirical data. A key limitation is that the models performance becomes worse as lesion size increases, leaving room for improvement in the future

I suggest the authors emphasise the results directly comparing the simulated FC edges to the

empirical FC edges (shown in the first paragraph of section 3.1). To me, this represents the most obvious test of model accuracy and in many ways should be what future papers should try to improve upon.
- In addition to correlation, could the authors report some measure of error magnitude (e.g., mean absolute error, MAE)?
- Can the authors quantify if the poorly predicted edges are the ones closest to a given individual's lesion site?

It appears as though the healthy data doesn’t contain the necessary information to predict larger perturbations to brain anatomy (e.g., the model performs worse in large lesions). Did the authors consider training a model that was fit to the empirical patient FC data? (and then tested in a held-out cohort). What additions to future models do they think will improve this limitation?

Minor:

I would suggest the authors add some statistics in the abstract to quantify their claims (perhaps the r, and MAE value between real and simulated FC).

Are the FC outcome measures interrelated (Intra / Inter and modularity)? Does the model predict each of these things independently or is it predicting a shared latent factor?

Why is section 2.4 not in 2.10? Perhaps the authors could make the distinction between these a little clearer.

Given the empirical FC is a poor predictor of behaviour, why would one expect the simulated FC to be a good predictor? If anything this analysis would suggest that the simulated FC contains some structural information not usually found in FC.

Figure 2 doesn’t outline all the abbreviations in the caption

Reviewer: 2

Comments to the Author
Overall, I think the models explored in the article are interesting to show what helps predict functional connectivity or not. However, the article is hard to follow and lacks clear details on the models besides mathematical considerations. The results are also confusing. This study, with some clarifications, can nonetheless benefit the scientific community.

Comments:
Introduction:
"In addition, the generative aspect of the model allows for generalization to new datasets." A model's generative ability does not imply generalization; it entirely depends on the training data and the nature of the new dataset. I think this sentence is incorrect and not really necessary.

"This generative, predictive whole-brain model, which satisfies an out-of-population validation criterion, differs from our previous stroke-related models since the latter were fit directly to the functional data of each patient." I think this sentence needs clarification. It is difficult to see the logical reasoning from the information presented so far.

Methods:
2.1: "single lesion stroke". Shouldn't this be one of the limitations as well? A large proportion of stroke patients have multiple lesions; would the model be able to deal with numerous foci?

The nature of the stroke lesions is only mentioned in the discussion; I think it would be helpful to know that the patients all have ischaemic strokes in this section.

2.2
I have never seen this method used to create an average tractography in a population. How representative of the anatomy is this average? Is it possible to find the same main white matter tracts as in individual tractography? How could this average affect the quality of the results leveraging its information compared with the individual data? Isn't it negating the interindividual variability?

2.4
"Modularity: overall Newman's modularity among cortical networks, a comparison between the number of connections within a module to the number of connections between modules". How is a module defined? Are the modules referring to the FC parcels?

2.6 I am surprised to see a 6-year-old article mentioned as state-of-the-art. I doubt there was no improvement to the methods to process fMRI and dMRI in 6 years.

"We computed the total amount of disconnection as a metric of anatomical impairment to assess the validity of the model." I don't understand. Is it the number of disconnected streamlines in the average tractography? As it is not mentioned, I am guessing that deterministic tractography was used; in this case, the number of streamlines is not a meaningful measure of connection (or disconnection) strength. Ten streamlines connecting the same regions could be disconnected. They would weigh more than five disconnected streamlines between five different couples of brain regions, even though they would more severely impact the symptoms.

2.8 "both scaled by a factor �� = 0.001" Why this scaling, and how was it chosen?

2.9 "First, we estimated the optimal value of global coupling for which the modeled Hilbert phases were most similar to the empirical data in the healthy controls group (G=0.75)." This is the first time the Hilbert phases are mentioned in the paper, and this has not been explained. What is it, and why should it be similar to the empirical data?

"As a result, the simulated time series (referred to as "Full Predictive model") contains structural information from the patient but not their functional information, making it a predictive model, in contrast to the previously discussed non-predictive models." I am not sure I understand what the authors mean by this sentence; maybe it should be clarified.

"Specifically, the healthy group-mean FC for a parcel pair was subtracted from the patient's FC for that parcel pair, and this difference score was then divided by the standard deviation of the healthy group FC for that parcel pair." How does the abnormality map difference compare with the variability present in the control dataset? As there is no reference to the amplitude of the

changes in the abnormality map, it is difficult to evaluate how much they differ.

"Where N is the set of all nodes in the network, n is the number of nodes, and (����) is a link between the nodes i and j." What is d_ij in the equation? The path length?

2.9.3: Even after reading the rest of the article, I do not understand the patient-specific model. I understand what information it contains, but I don't understand why or how it estimates anything.

Results
The results are very difficult to follow. It is not easy to figure out what has been compared or not. "1) a decrease of negative intra-hemispheric FC between regions of the Dorsal Attention Networks (DAN) and Default Mode Network (DMN); 2) a decrease of inter-hemispheric homotopic FC;" Isn't a decrease of negative an increase? If it is not meant this way, it needs clarification. These specific FCs have also been selected a priori, but I don't see these measures in other FCs or the global FC. It could also just mean that the model learned to apply the same change of FC across the brain, which would not be specific. Finally, I don't see the method used to compare these FCs. Is the difference statistically significant or just lower?

"Overall, the above results indicate that the Full Predictive model reproduced to some extent patient FC matrices, patient-specific abnormalities in the FC matrix, summary FC-based measures that are typically abnormal following a stroke, and patient-specific abnormalities in those measures." I do not see where the results show individual differences between patients. If it is there, it needs to be more explicit. Are the authors rather referring to changes specific to the patient population? If so, the sentence is ambiguous.

"The empirical FC matrix showed lower performance than the z-scored and simulated FC matrices across all domains, possibly because the predictive model that generated a patient's simulated FC matrix explicitly incorporated their structural disconnection matrix. Overall, these results indicate that although the predictive model partly accounted for behavioral abnormalities, the model did not generally perform better than a purely structural measure." This result is surprising. Multiple explanations come to mind that could tie to the previous result. I think it would be helpful to have the correlations between the different empirical FCs to compare how much of an individual patient's FC correlates with healthy FCs. This result could suggest that the structural information added to the model to predict the FC just incorporates this information without actually predicting a new FC. If the correlation between the group-level healthy FC and the empirical patient FC is comparable to that between the simulated FC and empirical FC, that would mean the structural information does not help predict the patient FC.

It seems that these analyses have been done in the supplementary material and confirm this.

The correlation between the amount of disconnection and the model's accuracy suggests this direction. It seems the more structural information, the further from empirical FC the simulated FC is. It looks like the structural information impairs the model's prediction. How are the predictions of the symptoms based on just the higher half of the patients in terms of disconnections? If this group is higher, it would suggest that the closer the simulated FC is to the SDC, the better the prediction, no?

Discussion:
"Adding lesion information to a model trained on healthy functional data is sufficient to reproduce functional anomalies in patient." Well, it is not sufficient, but it is potentially beneficial. If the prediction had been perfect, it would have been sufficient.

I think the discussion/conclusion should mention that the model needs improvement, considering its performance. It provides interesting insight into what information helps predict FC, but it would not be enough to reliably predict FC.

Comments from the Scientific Editorial team:

-Please add an Abbreviated Summary (up to 50 words) of your paper that captures the main purpose and conclusions of the work. The summary should state the main results written in the third person (‘[1st author surname] et al. report that ...’) and conclusion. This summary will be used in the contents list online and should not contain any abbreviations other than those on the accepted abbreviations list and accepted gene/protein names, making it accessible to the non-specialist. Please upload it as a Word document. Allowed abbreviations are: AIDS; ANOVA; AMPA; ATP; A,T,C,G; CNS; CSF; CT; DNA; ECG; EEG; EMG; GABA; HIV; MRI; NMDA; PET; PCR; RNA.

-Please provide a graphical abstract. A graphical abstract is a visual representation of the central finding or methodology of the paper. It will be displayed in the table of contents. Authors should strive to make the graphical abstract informative, interesting, visually appealing, and straightforward (no caption needed, no abbreviations except gene names). The technical requirements for the graphical abstracts are:
- Size: 120 x 120 mm (300-600dpi) or 3000 x 3000 pixels
- Font: Arial 12-16 pt
- File type: tiff or PDF
- Aspect ratio: 1:1 preferred

-Please include the appropriate checklist to comply with our ethical standards (see Reporting Guideline section within 'Instructions to authors').

-If applicable, please include a paragraph about statistical analysis in the 'Materials and Methods' section.

-Please provide the sex of the patients/subjects in the study.

-Figures 1, 2, 3 and 4, all supplementary figures: Please ensure all abbreviations used in the figure and figure legend are defined within the figure legend so that they may be easily interpreted as stand-alone figures.

-Figure 2, Supplementary figures 2, 3, 8 and 9: For each statistical analysis part of a figure, please report in the figure legend the details of the sample size (N=) used in the statistical test.

-Figure 3: If possible, arrange the figure panels/parts from left to right, then top to bottom.

-Figures 3, 4, Supplementary figure 10: For each statistical analysis part of a figure, please report in the figure legend the relevant test statistic values alongside p values (for example F or t values for ANOVAs and t-tests), and details of the sample size (N=).

-Figures 3F, 4C-D, Supplementary figures 5, 8: For these figures, if the sample size (N=) is <10, please show the single data-points.

-Figure 3B-D, Supplementary figure 2: Please revisit your colour choice in this figure as the green-red combination is not colour-blind friendly.

-Supplementary figures 5, 6 and 7: For each statistical analysis part of a figure, please report in the figure legend the statistical test used and details of the sample size (N=) used in the statistical test.

-As a reminder at this revision stage, if you plan to edit the current figures and tables, or to make new figures and tables, please ensure that these items fit a typeset journal page (A4), or are uploaded as supplementary material.
