# Supplementary material for: Generative whole-brain dynamics models from healthy subjects predict functional alterations in stroke at the level of individual patients
Source: Brain Commun. 2024 Jul 13;6(4):fcae237. doi: 10.1093/braincomms/fcae237 (PMC11285191; doi:10.1093/braincomms/fcae237)
Supplement: fcae237_Supplementary_Data [file Original_submission_Response_to_reviewers_comments.pdf › Original_submission_Response_to_reviewers_comments.pdf]

Dear Editor and Reviewers:

We thank you for the helpful feedback we have received on our manuscript. This letter contains our responses to each of the reviewers' comments. Our responses are provided below as indented paragraphs in blue, quotes or additions to the manuscript text are in green, and portions of the original text are in light green.

On behalf of all co-authors,

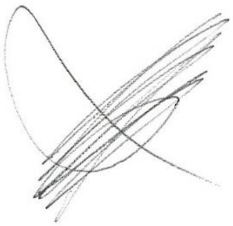

Sebastian Idesis, PhD.

Reviewer 1*: The current manuscript investigates the ability of a computational model to simulate the effect of stroke on fMRI functional connectivity from lesion maps, based on normative data. I think the research question is interesting, and has broad interest in the emerging field of "normative connectivity" modelling that is being used in stroke, as well as other disorders, like OCD. The authors show that their model is capable of reproducing several key phenomena that are observed in empirical data. A key limitation is that the models performance becomes worse as lesion size increases, leaving room for improvement in the future*

> We thank the reviewer for the encouraging comments.

Major:

1. *I suggest the authors emphasise the results directly comparing the simulated FC edges to the empirical FC edges (shown in the first paragraph of section 3.1). To me, this represents the most obvious test of model accuracy and in many ways should be what future papers should try to improve upon.*

> We have revised the text accordingly:

We first determined how well the model accounted for each subject's empirical FC matrix (healthy controls and stroke patients) by computing the Pearson correlation between the model-generated FC matrix and their empirical FC matrix. This analysis directly compares the similarity of the simulated FC edges to the empirical FC edges, as extensively reported in the literature (Deco et al., 2019; Idesis et al., 2023; Idesis, Faskowitz, et al., 2022).

2. *In addition to correlation, could the authors report some measure of error magnitude (e.g., mean absolute error, MAE)?*

We now report the Mean Squared Error. As examples, here are two modified sentences:

We found that higher model accuracy was associated with lower values of total structural disconnection (R: -.58, p<.01, MSE: 0.04)

Figure 2B shows that the empirical and simulated values for these three signatures of stroke were significantly correlated across patients (intra-hemispheric: r=.47, p<.01, MSE= .01; inter-hemispheric: r=.46, p<.01, MSE=.008; modularity: r=.40, p=.03, MSE=.07).

3. *Can the authors quantify if the poorly predicted edges are the ones closest to a given individual's lesion site?*

This is an interesting suggestion. We have added a panel to supplementary Figure 3, that shows (for a single patient) poorer model performance for edges closer to the patient's lesion site.

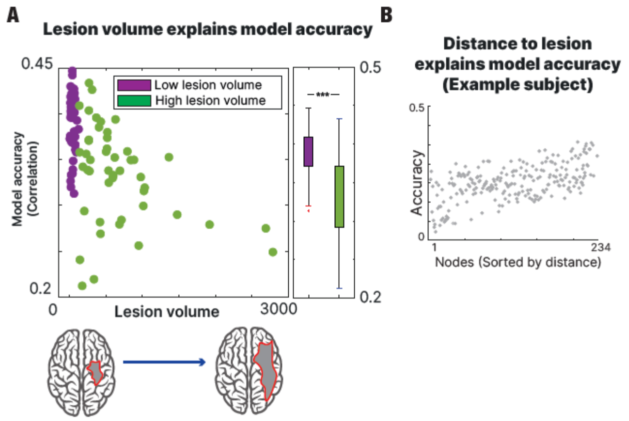

Supplementary Figure 3: Relation of lesion volume to model accuracy: (A) Patients with larger lesion volumes showed lower model accuracies (correlation between empirical and simulated data; N=96). (B) A scatterplot showing model accuracy for each node/region of interest as a function of its distance from the lesion site. Greater distances are associated with higher model accuracies (Single case example for visualization purposes).

4. *It appears as though the healthy data doesn't contain the necessary information to predict larger perturbations to brain anatomy (e.g., the model performs worse in large lesions). Did the authors consider training a model that was fit to the empirical patient FC data? (and then tested in a held-out cohort). What additions to future models do they think will improve this limitation?*

   We thank the reviewer for these suggestions and have added the paragraph below to the discussion section.

   Following this line, the presented model was trained with a healthy group population. Due to the heterogeneity of strokes, a bigger dataset would be needed to train the model directly with patient data. Specifically, a large amount of data would be needed to represent all stroke subgroups (different location, sizes, etc.) so that the model could be appropriately trained. Future studies could benefit from open-source datasets to achieve this goal.

Minor:

1. *I would suggest the authors add some statistics in the abstract to quantify their claims (perhaps the r, and MAE value between real and simulated FC).*

We have added the average correlation and Mean Square Error to the abstract:

We show that adding information about the structural disconnections produced by a patient's lesion to a whole-brain model previously trained on structural and functional data from a large cohort of healthy subjects predicts the resting functional connectivity of the patient about as well as fitting the model directly to the patient's data (Pearson Correlation =.37; Mean Square Error=.005).

2. *Are the FC outcome measures interrelated (Intra / Inter and modularity)? Does the model predict each of these things independently or is it predicting a shared latent factor?*

We thank the reviewer for this suggestion. We have performed an extra analysis showing the independence of the metrics in the model outcome in Supplementary figure 7B.

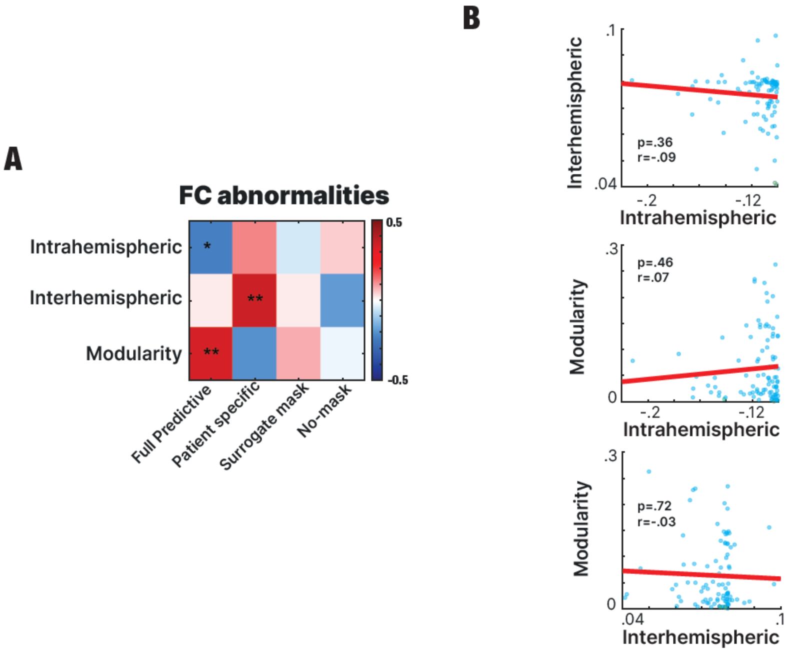

(B) We compared the three FC abnormalities calculated from the Full Predictive model and found no significant relation between them.

3. *Why is section 2.4 not in 2.10? Perhaps the authors could make the distinction between these a little clearer.*

   We have added text to the beginning of section 2.10 to make this distinction clearer

   To complement the typical metrics (See section 2.4) from the literature that assess functional brain dynamics after stroke (Adhikari et al., 2017; Corbetta et al., 2015; Siegel et al., 2016), we have included additional metrics of model accuracy:

4. *Given the empirical FC is a poor predictor of behaviour, why would one expect the simulated FC to be a good predictor? If anything this analysis would suggest that the simulated FC contains some structural information not usually found in FC.*

   We agree with the reviewer and discussed this interpretation of this result in Section 3.1, which noted that since the simulated FC is created by using the subjects' disconnection information, it incorporates some structural information:

   The empirical FC matrix showed lower performance than the z-scored and simulated FC matrices across all domains, possibly because the predictive model that generated a patient's simulated FC matrix explicitly incorporated their structural disconnection matrix.

5. *Figure 2 doesn't outline all the abbreviations in the caption*

   We have added the missing abbreviations in the caption to improve readability.

   (D) Separate partial least squares regression (PLSR) analyses were conducted using the empirical (FC EMP), z-scored (FC Z-ABN) and simulated FC (FCSIM) matrices from the Full Predictive model and the structural disconnection matrix (SDC) as regressors to predict each domain of behavioral impairment (Language; Motor Left; Motor Right; Attention-Visuospatial field bias; Attention-Average performance; Attention-Orientation to unattended stimuli; Memory spatial; Memory verbal; Motor independent component).

Reviewer 2*: Comments to the Author:*

Overall, I think the models explored in the article are interesting to show what helps predict functional connectivity or not. However, the article is hard to follow and lacks clear details on the models besides mathematical considerations. The results are also confusing. This study, with some clarifications, can nonetheless benefit the scientific community.

We thank the reviewer for the comments. We have added more details on the models and modified the text to make it clearer.

Introduction:

1. *"In addition, the generative aspect of the model allows for generalization to new datasets." A model's generative ability does not imply generalization; it entirely depends on the training data and the nature of the new dataset. I think this sentence is incorrect and not really necessary.*

   We have removed the aforementioned sentence.

2. *"This generative, predictive whole-brain model, which satisfies an out-of-population validation criterion, differs from our previous stroke-related models since the latter were fit directly to the functional data of each patient." I think this sentence needs clarification. It is difficult to see the logical reasoning from the information presented so far.*

   We have rephrased the sentence to make it clearer.

   Previous stroke-related models optimized the patient fitting by including both anatomical and functional information of each subject (Rocha et al., 2022). In contrast, this generative, predictive whole-brain model satisfies an out-of-population validation criterion, since it does not include any functional information from stroke patients.

Methods:

2.1: "single lesion stroke". Shouldn't this be one of the limitations as well? A large proportion of stroke patients have multiple lesions; would the model be able to deal with numerous foci?

We thank the reviewer for this comment and have added the corresponding sentence to the limitations section.

Finally, the presented dataset consisted mostly of single lesion stroke patients. Future studies could evaluate model accuracy for patients with multiple lesions.

The nature of the stroke lesions is only mentioned in the discussion; I think it would be helpful to know that the patients all have ischaemic strokes in this section.

We thank the reviewer for this comment. We added the corresponding clarification at the end of the mentioned sentence.

We used the Washington University Stroke Cohort dataset (Corbetta et al., 2015), a large prospective longitudinal (two weeks, three months, 12 months) study of patients with a first-time, single lesion stroke (83% ischemic, 17% hemorrhagic).

2.2

I have never seen this method used to create an average tractography in a population. How representative of the anatomy is this average? Is it possible to find the same main white matter tracts as in individual tractography? How could this average affect the quality of the results leveraging its information compared with the individual data? Isn't it negating the interindividual variability?

The Yeh atlas has been used previously as a representation of the healthy adult structural connectome (e.g. Griffis, 2019, 2021), but we acknowledge that individual differences in the structural connectome of a patient pre-stroke will presumably affect the accuracy for predicting the patient's FC post-stroke and limit overall model accuracy. We have added the following text to the manuscript:

The predictive patient model presented in this study is based partly on a group structural representation, i.e. a group healthy structural connectome, and partly on patient-specific structural information i.e. the patient's lesion. As such, inaccuracies in how well the group healthy connectome corresponds to a patient's pre-stroke structural connectome will affect the accuracy of the model for that patient. Therefore, it is relevant to clarify that for stroke patients, inter-

individual variability in structure is dominated by the disconnection due to the lesion, rather than fine-grained individual differences in the pre-stroke connectome.

2.4

"Modularity: overall Newman's modularity among cortical networks, a comparison between the number of connections within a module to the number of connections between modules". How is a module defined? Are the modules referring to the FC parcels?

We have extended the definition of modules to increase the understanding of the concept

Modularity: overall Newman's modularity among cortical networks, a comparison between the number of connections within a module to the number of connections between modules. Here, by 'module' we mean what is commonly called 'module' or 'community' or 'cluster' in network theory, i.e., a set of nodes forming a tightly-knit subnetwork (in this case, modules correspond to major resting state networks).

2.6 I am surprised to see a 6-year-old article mentioned as state-of-the-art. I doubt there was no improvement to the methods to process fMRI and dMRI in 6 years.

We have removed the "state-of-the-art" statement from the 2.6.1 section.

"We computed the total amount of disconnection as a metric of anatomical impairment to assess the validity of the model." I don't understand. Is it the number of disconnected streamlines in the average tractography? As it is not mentioned, I am guessing that deterministic tractography was used; in this case, the number of streamlines is not a meaningful measure of connection (or disconnection) strength. Ten streamlines connecting the same regions could be disconnected. They would weigh more than five disconnected streamlines between five different couples of brain regions, even though they would more severely impact the symptoms.

We thank the reviewer for bringing up this issue. We have added a sentence to this section to explain more in detail this metric and to provide the missing references.

For each patient, region-based disconnections were binarized at a 1% disconnection threshold. A total disconnection score for a patient was then determined by summing the number of disconnected regions. Therefore, the disconnection is obtained from each subject SDC for which

the relevance is driven but the amount and not the location or consequences of the lesion, as reported in previous literature (Griffis et al., 2019, 2021; Idesis, Favaretto, et al., 2022).

2.8 "both scaled by a factor �� = 0.001" Why this scaling, and how was it chosen?

We have modified the sentence to clarify the source of this factor's value:

as reported extensively for optimization through a gradient-descent approach in previous literature (Deco et al., 2023; Idesis, Favaretto, et al., 2022; Perl et al., 2023)

2.9 "First, we estimated the optimal value of global coupling for which

the modeled Hilbert phases were most similar to the empirical data in the healthy controls group (G=0.75)." This is the first time the Hilbert phases are mentioned in the paper, and this has not been explained. What is it, and why should it be similar to the empirical data?

We added a sentence clarifying the concept of Hilbert phases and the reason it was used:

First, the average BOLD time series of each region of interest were Hilbert-transformed to yield the phase evolution of the regional signals as reported in previous literature as a proxy for brain dynamics similarity (Deco et al., 2019; Idesis, Favaretto, et al., 2022). Then, we estimated the optimal value of global coupling for which the modeled Hilbert phases were most similar to the empirical data in the healthy controls group (G=0.75).

"As a result, the simulated time series (referred to as "Full Predictive model") contains structural information from the patient but not their functional information, making it a predictive model, in contrast to the previously discussed non-predictive models." I am not sure I understand what the authors mean by this sentence; maybe it should be clarified.

We have rephrased the corresponding sentence to make it easier to read.

Previous models included both structural and functional information from each stroke patient, making them "non-predictive" models. In contrast, the "Full predictive Model" used to generate simulated time series contained structural information from the patient but not their functional information, making it a predictive model.

"Specifically, the healthy group-mean FC for a parcel pair was subtracted from the patient's FC for that parcel pair, and this difference score was then divided by the standard deviation of the

healthy group FC for that parcel pair." How does the abnormality map difference compare with the variability present in the control dataset? As there is no reference to the amplitude of the changes in the abnormality map, it is difficult to evaluate how much they differ.

We have included the mean and standard deviation of the matrices in order to quantify the empirical data in healthy controls and stroke patients, next to the simulated matrices obtained by the predictive model in stroke subjects.

To assess whether the Full Predictive model accurately predicted specifically the abnormalities in a patient's FC that resulted from their stroke, we correlated each patient's empirical and simulated z-scored FC abnormality matrices. We performed this analysis by normalizing the matrix with the healthy control group (Healthy FC: mean = .12, std=.02; Z-Empirical: mean=.046, std=.01; Z-Simulated: mean=.039, std=.01).

"Where N is the set of all nodes in the network, n is the number of nodes, and (����) is a link between the nodes i and j." What is d_ij in the equation? The path length?

We added a sentence clarifying all the terms in the equation

Where N is the set of all nodes in the network, n is the number of nodes, and (d_{ij}) is the shortest path length between the nodes i and j.

2.9.3: Even after reading the rest of the article, I do not understand the patient-specific model. I understand what information it contains, but I don't understand why or how it estimates anything.

We apologize to the reviewer for the lack of clarity. We explained it in a further figure (Figure 4A) to support the explanation of the model

The patient-specific model used each patient's own functional information (i.e., their BOLD timeseries) as well as their structural disconnection matrix to estimate the parameters of their model, making the model non-predictive. By including both anatomical and functional information, the patient-specific model could be used to predict the effect of perturbations in the system, such as stimulation.

Results

The results are very difficult to follow. It is not easy to figure out what has been compared or not.

"1) a decrease of negative intra-hemispheric FC between regions of the Dorsal Attention Networks (DAN) and Default Mode Network (DMN); 2) a decrease of inter-hemispheric homotopic FC;" Isn't a decrease of negative an increase? If it is not meant this way, it needs clarification. These specific FCs have also been selected a priori, but I don't see these measures in other FCs or the global FC. It could also just mean that the model learned to apply the same change of FC across the brain, which would not be specific. Finally, I don't see the method used to compare these FCs. Is the difference statistically significant or just lower?

First, indeed a decrease of a negative is an increase but we chose that way to express the results to be consistent with previous literature in stroke research that report their findings in that way (Adhikari et al., 2017; Griffis et al., 2019; Siegel et al., 2017; Siegel et al., 2016). Regarding the FCs, we have added text to clarify the chosen method and metrics:

When exploring the levels of intra-hemispheric connectivity, we focused on the connectivity between DAN and DMN, as this comparison has been used extensively in the literature (Adhikari et al., 2021; Corbetta et al., 2018; Griffis et al., 2019; Idesis, Favaretto, et al., 2022; Siegel et al., 2017; Siegel et al., 2016; Siegel et al., 2022). As this metric is not performed globally but in specific regions, we complemented it with other whole-brain metrics such as inter-hemispheric homotopic FC level and modularity. Furthermore, we have also included a diverse set of functional dynamic metrics that capture the global impact of stroke lesions (See section 2.10).

"Overall, the above results indicate that the Full Predictive model reproduced to some extent patient FC matrices, patient-specific abnormalities in the FC matrix, summary FC-based measures that are typically abnormal following a stroke, and patient-specific abnormalities in those measures." I do not see where the results show individual differences between patients. If it is there, it needs to be more explicit. Are the authors rather referring to changes specific to the patient population? If so, the sentence is ambiguous.

We use the term 'patient-specific' because the model is separately computed for each individual patient. The accuracy of the model in predicting measures of FC for an individual patient is shown by the correlation coefficient between the empirical and simulated FC measures for that patient, and these single-patient correlations are plotted in the figures (e.g. Figure 2D). The reviewer is correct that we do not statistically compare different patients, although since patients can have very different lesions, both in topography and volume, individual differences are expected.

"The empirical FC matrix showed lower performance than the z-scored and simulated FC matrices across all domains, possibly because the predictive model that generated a patient's simulated FC matrix explicitly incorporated their structural disconnection matrix. Overall, these results indicate that although the predictive model partly accounted for behavioral abnormalities, the model did not generally perform better than a purely structural measure." This result is surprising. Multiple explanations come to mind that could tie to the previous result. I think it would be helpful to have the correlations between the different empirical FCs to compare how much of an individual patient's FC correlates with healthy FCs. This result could suggest that the structural information added to the model to predict the FC just incorporates this information without actually predicting a new FC. If the correlation between the group-level healthy FC and the empirical patient FC is comparable to that between the simulated FC and empirical FC, that would mean the structural information does not help predict the patient FC. It seems that these analyses have been done in the supplementary material and confirm this.

We have addressed this issue in supplementary figure 10, which displays the influence of the GEC in the model (i.e. the incorporation of a model optimization based on the healthy controls' functional data). The accuracy of a patient model in predicting their FC was significantly higher when the model was based on a healthy control model that specifically incorporated GEC. This result shows that model optimization significantly increases accuracy above what is expected based on structural information. Previous studies have analyzed the influence of the structural information in the performance of whole-brain models and shown their key importance (Idesis, Favaretto, et al., 2022; Rocha et al., 2022; Siegel et al., 2022)

The correlation between the amount of disconnection and the model's accuracy suggests this direction. It seems the more structural information, the further from empirical FC the simulated FC is. It looks like the structural information impairs the model's prediction. How are the predictions of the symptoms based on just the higher half of the patients in terms of disconnections? If this group is higher, it would suggest that the closer the simulated FC is to the SDC, the better the prediction, no?

Figure 3A and supplementary Figure 3 show the poorer performance of the model for the higher half of the patients with the most disconnection/damage. We suggest that the structural information per se does not impair the accuracy of the model. Rather, model accuracy is impaired when there

is a great deal of damage. In support of this idea, the inclusion of structural information concerning the healthy connectome in whole-brain computational models increases the accuracy of those models in predicting the FC of healthy subjects, i.e. the inclusion of structural information increases model performance over what would be found if that information were not included. *Discussion:*

"Adding lesion information to a model trained on healthy functional data is sufficient to reproduce functional anomalies in patient." Well, it is not sufficient, but it is potentially beneficial. If the prediction had been perfect, it would have been sufficient.

We agree with the reviewer and have modified the sentence.

Adding lesion information to a model trained on healthy functional data is beneficial to reproduce functional anomalies in patients.

I think the discussion/conclusion should mention that the model needs improvement, considering its performance. It provides interesting insight into what information helps predict FC, but it would not be enough to reliably predict FC.

We agree with the reviewer and have added the following text to the discussion section:

Given the moderate accuracy of the model, as well as the decrease in model accuracy as the damage from a stroke increases, we acknowledge there is room for improvement in future work, which could include the application of the model to other focal and non-focal pathologies that damage the structural connectome.

Comments from the Scientific Editorial team:

-Please add an Abbreviated Summary (up to 50 words) of your paper that captures the main purpose and conclusions of the work. The summary should state the main results written in the third person ('[1st author surname] et al. report that …') and conclusion. This summary will be used in the contents list online and should not contain any abbreviations other than those on the accepted abbreviations list and accepted gene/protein names, making it accessible to the non-specialist. Please upload it as a Word document. Allowed abbreviations are: AIDS; ANOVA; AMPA; ATP; A,T,C,G; CNS; CSF; CT; DNA; ECG; EEG; EMG; GABA; HIV; MRI; NMDA; PET; PCR; RNA.

We have incorporated an Abbreviated abstract below the already existing abstract:

Abbreviated abstract:

Idesis et al. show that functional anomalies in stroke patients can be predicted mechanistically by adding only structural information concerning their lesion to a whole-brain computational model computed solely from healthy controls. Their results validate the integration of structural and functional information in whole-brain models of stroke.

-Please provide a graphical abstract. A graphical abstract is a visual representation of the central finding or methodology of the paper. It will be displayed in the table of contents. Authors should strive to make the graphical abstract informative, interesting, visually appealing, and straightforward (no caption needed, no abbreviations except gene names). The technical requirements for the graphical abstracts are:

- Size: 120 x 120 mm (300-600dpi) or 3000 x 3000 pixels
- Font: Arial 12-16 pt
- File type: tiff or PDF
- Aspect ratio: 1:1 preferred

We have added the corresponding graphical abstract:

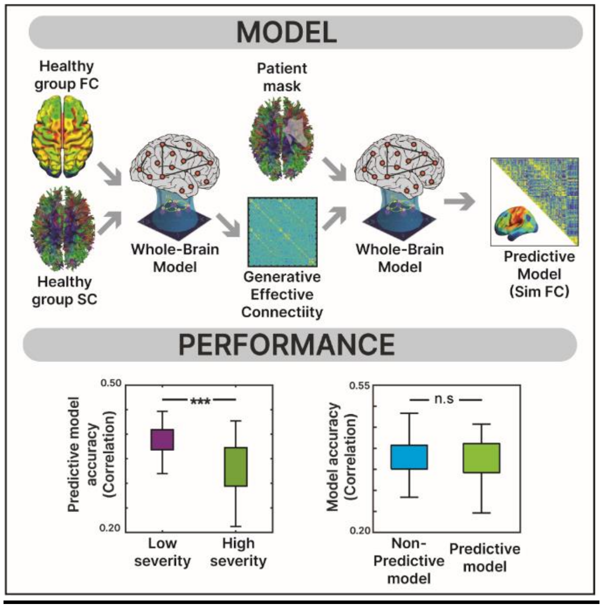

-Please include the appropriate checklist to comply with our ethical standards (see Reporting Guideline section within 'Instructions to authors').

As discussed previously with the journal, out study uses a widely published dataset for which there is no need to include the corresponding checklist.

-If applicable, please include a paragraph about statistical analysis in the 'Materials and Methods' section.

We considered that a paragraph about statistical analysis is not necessary as the statistical approaches used for each result is reported in each corresponding section.

-Please provide the sex of the patients/subjects in the study

We added the sex of the subjects in Section 2.1

From this cohort, we selected 96 stroke patients (M=57%, F=43%) and 27 healthy subjects (M=59%, F=41%).

-Figures 1, 2, 3 and 4, all supplementary figures: Please ensure all abbreviations used in the figure and figure legend are defined within the figure legend so that they may be easily interpreted as stand-alone figures.

We have added to each figure legend the missing abbreviations.

-Figure 2, Supplementary figures 2, 3, 8 and 9: For each statistical analysis part of a figure, please report in the figure legend the details of the sample size (N=) used in the statistical test.

We have added in each figure legend the missing information.

Figure 2:

Figure 2: Model prediction of patient FC and behavior. (A) The Pearson correlation between the empirical and simulated FC matrices of each subject (27 healthy controls and 96 stroke patients) was computed in order to assess the accuracy of the models.

Supp. Figure 2:

Then the simulated and empirical averaged quantities were plotted against one another across patients (N=96).

Supp. Figure 3

Patients with larger lesion volumes showed lower model accuracies (correlation between empirical and simulated data; N=96).

Supp. Figure 8

FCD was estimated for the empirical data and the three predictive models showing a significant difference between the predictive models and the model without mask (N=96).

Supp. Figure 9

Subjects with lower levels of disconnection exhibited a higher correlation between the empirical and simulated FC matrices, indicating better model performance for patients with less severe lesions (N=96).

-Figure 3: If possible, arrange the figure panels/parts from left to right, then top to bottom.

We have re-arranged the panels to be read as requested

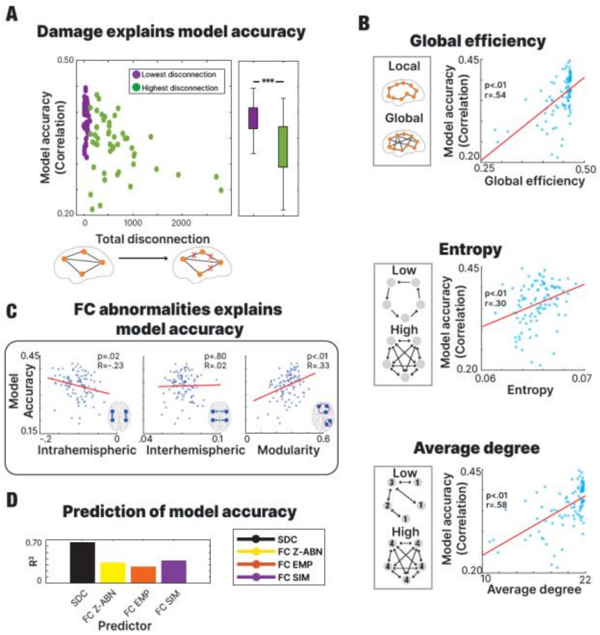

-Figures 3, 4, Supplementary figure 10: For each statistical analysis part of a figure, please report in the figure legend the relevant test statistic values alongside p values (for example F or t values for ANOVAs and t-tests), and details of the sample size (N=).

We have added in each figure legend the missing information.

Figure 3:

(A) Subjects with lower levels of disconnection exhibited a higher correlation between the empirical and simulated FC matrices, indicating better model performance for patients with less severe lesions ($t(94)= 5.82$, $p< .01$; N=96).

(F) Model accuracy predicted by each type of regressor (SDC matrix, , z-scored FC abnormality matrix, FC empirical and FC simulated matrices) in a PLSR analysis of model accuracy (N=96).

Figure 4:

(C) The similarity between the empirical and simulated FC matrices was assessed for each model. The non-predictive patient specific model and full predictive model showed similar levels of performance that exceeded performance for the surrogate and no-mask models ($F(3,285)= 14.84$, $p<.01$; N=96). (D) Comparison of model performance when dividing by disconnected nodes and directly connected nodes showed a significant interaction between model and node connection ($F(1, 190) = 67.96$, $p < .01$; N=96).

Supplementary figure 10:

The GEC showed a significantly higher performance showing the relevance of model optimization ($t(95)= 10.22$, $p< .01$; N=96).

-Figures 3F, 4C-D, Supplementary figures 5, 8: For these figures, if the sample size (N=) is <10, please show the single data-points.

The figures bar/box plots are made from the 96 stroke patients and therefore the sample size is greater than 10.

-Figure 3B-D, Supplementary figure 2: Please revisit your colour choice in this figure as the green-red combination is not colour-blind friendly.

We have updated our figures to have a different color combination more color-blind friendly

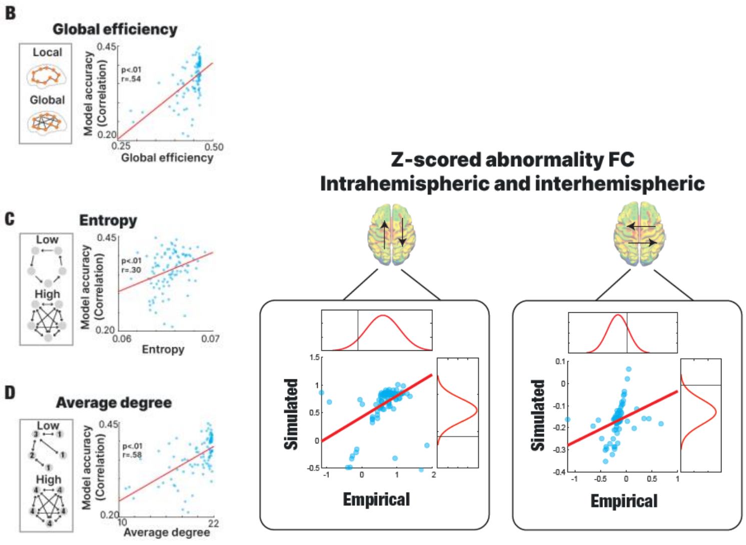

-Supplementary figures 5, 6 and 7: For each statistical analysis part of a figure, please report in the figure legend the statistical test used and details of the sample size (N=) used in the statistical test.

We have added the requested information in the figure legends:

Supplementary Figure 5:

Through an ANOVA, we observed that there was a significant difference between the group (F(4,406)= 27.84, p<.01) as the healthy control model showed the best accuracy and was included to assess the influence of the stroke lesion on model accuracy (N controls = 27, N patients = 96).

Supplementary Figure 6:

The similarity between the (A) empirical and (B) simulated data was assessed with different values of global coupling to determine the optimal point for the simulations. The chosen optimal G value was obtained as the one with the lowest error (G=.75) averaged across all healthy subjects (N=27).

Supplementary Figure 7:

The accuracy of the patient specific model was significantly correlated (Through Pearson Correlation) with inter-hemispheric FC (R=.41, p<.01), but not with intra-hemispheric FC or modularity. All the other models (including all the possibilities of the surrogate mask model and the No-mask model), showed no significant relationship with FC abnormalities (N=96).

-As a reminder at this revision stage, if you plan to edit the current figures and tables, or to make new figures and tables, please ensure that these items fit a typeset journal page (A4), or are uploaded as supplementary material.

We thank the editors for the reminder

Adhikari, M. H., Griffis, J., Siegel, J. S., Thiebaut de Schotten, M., Deco, G., Instabato, A., Gilson, M., & Corbetta, M. (2021). Effective connectivity extracts clinically relevant prognostic information from resting state activity in stroke. *Brain communications*, *3*(4), fcab233.

Adhikari, M. H., Hacker, C. D., Siegel, J. S., Griffa, A., Hagmann, P., Deco, G., & Corbetta, M. (2017). Decreased integration and information capacity in stroke measured by whole brain models of resting state activity. *Brain*, *140*(4), 1068-1085.

Corbetta, M., Ramsey, L., Callejas, A., Baldassarre, A., Hacker, C. D., Siegel, J. S., Astafiev, S. V., Rengachary, J., Zinn, K., & Lang, C. E. (2015). Common behavioral clusters and subcortical anatomy in stroke. *Neuron*, *85*(5), 927-941.

Corbetta, M., Siegel, J. S., & Shulman, G. L. (2018). On the low dimensionality of behavioral deficits and alterations of brain network connectivity after focal injury. *Cortex*, *107*, 229-237.

Deco, G., Cruzat, J., Cabral, J., Tagliazucchi, E., Laufs, H., Logothetis, N. K., & Kringelbach, M. L. (2019). Awakening: Predicting external stimulation to force transitions between different brain states. *Proceedings of the National Academy of Sciences*, *116*(36), 18088-18097.

Deco, G., Lynn, C. W., Sanz Perl, Y., & Kringelbach, M. L. (2023). Violations of the fluctuation-dissipation theorem reveal distinct nonequilibrium dynamics of brain states. *Physical Review E*, *108*(6), 064410. https://doi.org/10.1103/PhysRevE.108.064410

Griffis, J. C., Metcalf, N. V., Corbetta, M., & Shulman, G. L. (2019). Structural Disconnections Explain Brain Network Dysfunction after Stroke. *Cell Rep*, *28*(10), 2527-2540 e2529. https://doi.org/10.1016/j.celrep.2019.07.100

Griffis, J. C., Metcalf, N. V., Corbetta, M., & Shulman, G. L. (2021). Lesion Quantification Toolkit: A MATLAB software tool for estimating grey matter damage and white matter

disconnections in patients with focal brain lesions. *Neuroimage Clin*, *30*, 102639. https://doi.org/10.1016/j.nicl.2021.102639

Idesis, S., Allegra, M., Vohryzek, J., Sanz Perl, Y., Faskowitz, J., Sporns, O., Corbetta, M., & Deco, G. (2023). A low dimensional embedding of brain dynamics enhances diagnostic accuracy and behavioral prediction in stroke. *Scientific Reports*, *13*(1), 1-17.

Idesis, S., Faskowitz, J., Betzel, R. F., Corbetta, M., Sporns, O., & Deco, G. (2022). Edge-centric analysis of stroke patients: An alternative approach for biomarkers of lesion recovery. *NeuroImage: Clinical*, *35*, 103055.

Idesis, S., Favaretto, C., Metcalf, N. V., Griffis, J. C., Shulman, G. L., Corbetta, M., & Deco, G. (2022). Inferring the dynamical effects of stroke lesions through whole-brain modeling. *NeuroImage: Clinical*, *36*, 103233.

Perl, Y. S., Geli, S., Perez-Ordoyo, E., Zonca, L., Idesis, S., Vohryzek, J., Jirsa, V. K., Kringelbach, M. L., Tagliazucchi, E., & Deco, G. (2023). Whole-brain modelling of low-dimensional manifold modes reveals organising principle of brain dynamics. *BioRxiv*, 2023.2011.2020.567824. https://doi.org/10.1101/2023.11.20.567824

Rocha, R. P., Koçillari, L., Suweis, S., De Filippo De Grazia, M., de Schotten, M. T., Zorzi, M., & Corbetta, M. (2022). Recovery of neural dynamics criticality in personalized whole-brain models of stroke. *Nature Communications*, *13*(1), 3683.

Siegel, J. S., Mitra, A., Laumann, T. O., Seitzman, B. A., Raichle, M., Corbetta, M., & Snyder, A. Z. (2017). Data quality influences observed links between functional connectivity and behavior. *Cerebral cortex*, *27*(9), 4492-4502.

Siegel, J. S., Ramsey, L. E., Snyder, A. Z., Metcalf, N. V., Chacko, R. V., Weinberger, K., Baldassarre, A., Hacker, C. D., Shulman, G. L., & Corbetta, M. (2016). Disruptions of network connectivity predict impairment in multiple behavioral domains after stroke. *Proc Natl Acad Sci U S A*, *113*(30), E4367-4376. https://doi.org/10.1073/pnas.1521083113

Siegel, J. S., Shulman, G. L., & Corbetta, M. (2022). Mapping correlated neurological deficits after stroke to distributed brain networks. *Brain Structure and Function*, *227*(9), 3173-3187.
