# Supplementary material for: Generative whole-brain dynamics models from healthy subjects predict functional alterations in stroke at the level of individual patients
Source: Brain Commun. 2024 Jul 13;6(4):fcae237. doi: 10.1093/braincomms/fcae237 (PMC11285191; doi:10.1093/braincomms/fcae237)
Supplement: fcae237_Supplementary_Data [file Revision_1_ Response_to_reviewers_comments.pdf › Revision_1_ Response_to_reviewers_comments.pdf]

Dear Editor and Reviewers:

We thank you for the helpful feedback we have received on our manuscript. This letter contains our responses to each of the reviewers' comments. Our responses are provided below as indented paragraphs in blue, quotes or additions to the manuscript text are in green, and portions of the original text are in light green.

On behalf of all co-authors,

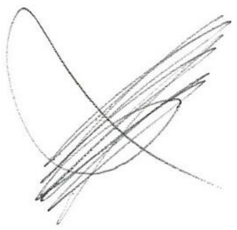

Sebastian Idesis, PhD.

Reviewer: 1

Comments to the Author

The Authors have responded to each of my queries and suggestions - I have no further feedback.

We thank the reviewer for the contribution. We believe the suggestions improved the quality of the manuscript

Reviewer: 2

Comments to the Author

The authors mostly replied to all my comments.

I am still a bit confused with the increase of structural disconnection leading to a lower performance and maybe it could do with a comment on that in the paper. But, besides that, I think the paper can be accepted in that revised form.

We appreciate the feedback of the reviewer. We have extended this topic explanation in the discussion section to increase clarity.

Moreover, the accuracy of the Full Predictive model depended on incorporating the structural disconnection specific to that patient's lesion, as shown by the significantly poorer performance obtained by substituting the structural disconnection for a different patient. This derived from the fact that a higher level of structural disconnection (more severe lesions) deviates more from the trained sample (consisted in healthy controls).

Comment from the Scientific Editor:

- *during the next revision stage, if you plan to edit the current figures and tables or make new ones, please ensure that these items fit on an A4 page.*

We modified the corresponding figures as requested by the editor

- *Please include the appropriate checklist to comply with our ethical standards (see Reporting Guideline section within 'Instructions to authors').*

The checklist is not needed as reported through email by the journal's editorial committee.

- *thank you for providing a graphical abstract, but please spell out SC and FC.*

We have updated the graphical abstract to avoid abbreviations.

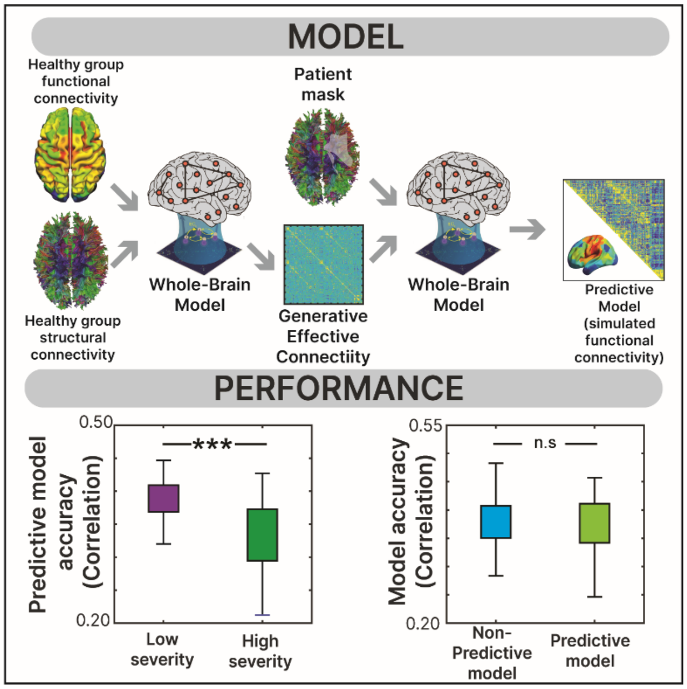

-Please include a paragraph about statistical analysis in the 'Materials and Methods' section.

We have included the requested paragraph in the "Materials and Methods" section

Statistical analysis

We performed paired/unpaired parametrical statistical analyses (t-test/ANOVA, based on the group number) depending on if the comparison was performed for the same patient or between subjects. Statistical significance was assessed with a threshold of p=.05. All *post hoc* comparisons were Bonferroni corrected for the number of comparisons taking place.

- *Fig1: please spell out SC in the corresponding legend.*

We have written the complete concept name to avoid unexplained abbreviations

Figure 1: Pipeline for the predictive model: (A) Healthy control generative effective connectivity (GEC) was calculated by using the healthy template structural connectivity (SC) with each healthy control fMRI time series. The model was optimized using a whole-brain (WB) model to create an average GEC for the healthy controls. **(B)** The predictive model used each patient's disconnection mask to modify the control GEC and obtain the patient's simulated functional connectivity (FC),

- *Fig2a and SuppFig3a: please report in the figure legend the relevant test statistic values alongside p values (for examsple F or t values for ANOVAs and t-tests).*

We have added the relevant test statistic values to figure 2a and Supp Fig3a:

Figure 2: Model prediction of patient FC and behavior. (A) The Pearson correlation between the empirical and simulated FC matrices of each subject (27 healthy controls and 96 stroke patients) was computed in order to assess the accuracy of the models (healthy controls: r=.43; stroke patients: r=.37).

Supplementary Figure 3: Relation of lesion volume to model accuracy: **(A)** Patients with larger lesion volumes showed lower model accuracies (correlation between empirical and simulated data; N=96). This was revealed when performing a t-test analysis (t(94)= 6.08, p< .001).

- *Fig2a: please, label the x axis and move the line hiding the x- axis numbers.*

We have modified the figure as requested:

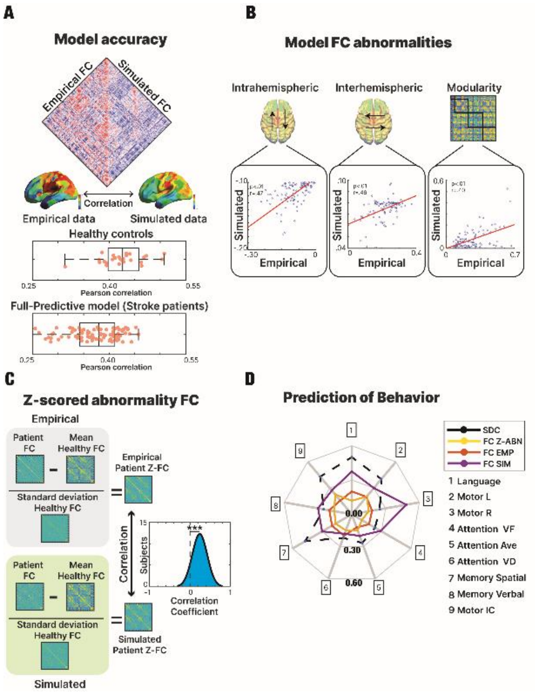

- *Fig3e-f: please, mention in the legend the two subfigures.*

As there is no subpanel e to f (modified after the first submission), the legend correspond accordingly to the uploaded figure

(B) Higher global efficiency, entropy, and average degree were associated with higher model accuracy.

- *Fig4c-d, Supp Fig3a and Supp Fig6: please report in the figure legend the test used.*

We clarified the test used in each corresponding figure:

Figure. 4: Model comparisons: (C) The similarity between the empirical and simulated FC matrices was assessed for each model. A within-subject ANOVA indicated that the non-predictive patient specific model and full predictive model showed similar levels of performance that exceeded performance for the surrogate and no-mask models ($F(3,285)= 14.84$, $p<.01$; N=96). (D) A two-way ANOVA indicated that the comparison of model performance when dividing by disconnected nodes and directly connected nodes showed a significant interaction between model and node connection ($F(1, 190) = 67.96$, $p < .01$; N=96).

Supplementary Figure 3: Relation of lesion volume to model accuracy: **(A)** Patients with larger lesion volumes showed lower model accuracies (correlation between empirical and simulated data; N=96). This was revealed when performing a t-test analysis ($t(94)= 6.08$, $p< .001$).

Supplementary Figure 6: Influence of global coupling (GC): The similarity between the **(A)** empirical and **(B)** simulated data was assessed with different values of global coupling to determine the optimal point for the simulations. The chosen optimal G parameter value was obtained as the one with the lowest error (G=.75) averaged across all healthy subjects (N=27). No statistical test was used for this selection.

- *Our system identified a high degree of similarity between portions of your manuscript, in particular the methods section, and previously published papers. Please, edit the manuscript to reduce such similarity, using the attached report as a reference.*

We thank the editorial team for the comment. We have modified substantially the methods section to reduce the similarity with previous publications.
