# Supplementary material for: Generative whole-brain dynamics models from healthy subjects predict functional alterations in stroke at the level of individual patients
Source: Brain Commun. 2024 Jul 13;6(4):fcae237. doi: 10.1093/braincomms/fcae237 (PMC11285191; doi:10.1093/braincomms/fcae237)
Supplement: fcae237_Supplementary_Data [file Revision_2_decision_letter.pdf › Revision_2_decision_letter.pdf]

**From:** brcoms.editorialoffice@oup.com
**To:**
**CC:**
**BCC:**
**Subject:** Brain Communications - Decision on Manuscript ID BRAINCOM-2024-030.R2
**Body:** 15-May-2024

Dear Dr. Idesis,

Pending the upload of final files according to our formatting requirements for production, it will be a pleasure to accept your manuscript entitled "Generative whole-brain dynamics models from healthy subjects predict functional alterations in stroke at the level of individual patients." for publication in Brain Communications.  Any comments made by the reviewer(s) are included below.

The next step is for your paper to go through “First Look”. We will contact you again shortly if any final files or formatting changes are required for final acceptance for publication, or if we have any queries.

If no changes are needed, your submission will be transferred to our Production Department and you will hear from the Production Editor in due course.

VIDEO ABSTRACTS - Brain Communications encourages authors to provide a video abstract to accompany their article. These are hosted on the Brain/Brain Communications YouTube channel. If you are interested in producing a 5-min video abstract to promote your article, then please contact the Editorial Office (brcoms.editorialoffice@oup.com) for further details and guidelines. (We don't currently accept video abstracts for Scientific Commentaries and Letters to the Editor).

PRESS RELEASES: If you are interested in coordinating the timing of any press releases or publicity with the publication of your article it may be useful to agree an embargo date. Please let us know in the Editorial Office (brcoms.editorialoffice@oup.com) as soon as possible so that we can start to put arrangements in place before we send your article to the Production Team.

We are always grateful to receive suggestions for cover images for the journal, based on the content of your paper. Please contact the Editorial Office for further information, at brcoms.editorialoffice@oup.com.

Thank you very much for your contribution.  On behalf of the Editors of Brain Communications, we look forward to your future contributions to the Journal.

Best wishes

Dr. Manuela Marescotti
Scientific Editor, Brain Communications

Professor Tara Spires-Jones
Founding Editor, Brain Communications
